# Supplementary material for: Ribosomal RNA operons define a central functional compartment in the Streptomyces chromosome
Source: Nucleic Acids Res. 2022 Nov 21;50(20):11654–69. doi: 10.1093/nar/gkac1076 (PMC9723626; doi:10.1093/nar/gkac1076)
Supplement: gkac1076_Supplemental_Files [file gkac1076_supplemental_files.zip › Supplementary material_list_v2.pdf]

Supplementary material available at NAR online

Supplementary Figure S1: Proposed *rrn* nomenclature based on core gene environments

Supplementary Figure S2: Pairwise comparisons of all the core genomes of the panel with the consensus reference strain, *Streptomyces viridosporus* T7A ATCC 39115 (15 pages)

Supplementary Figure S3: Pairwise comparison of the core genomes that are proposed to share rearrangements that occurred in their common ancestor (3 pages)

Supplementary Figure S4: Generalized linear model of the core region size based on the knowledge of the *rrn* operons (number, locations), the chromosome size and the *oriC* position

Supplementary Figure S5: Gene persistence along the chromosomes of 127 *Streptomyces* strains/species (15 pages)

Supplementary Figure S6: Mean gene persistence in the regions surrounding the *rrn* operons

Supplementary Figure S7: GO enrichment analysis of the core genes which are mainly located in the central compartment (A) or in the terminal compartments (B)

Supplementary Figure S8: Level of gene persistence and expression along the chromosome of *Streptomyces* species of interest

Supplementary Figure S9: Level of gene expression over growth depending on gene category (core, non-core or SMBGCs) and location inside or outside the central compartment

Supplementary Table S1: Genomic features of the *Streptomyces* genomes analyzed in this study

Supplementary Table S2: Annotation of all *Streptomyces* genomes analyzed in this study

Supplementary Table S3: ANIb values calculated between all species of the panel

Supplementary Table S4: Position of large genome rearrangements within the central compartment of *Streptomyces* from the panel of interest

Supplementary Table S5: Core genome annotation of *Streptomyces coelicolor* A3(2)

Supplementary Table S6: DESeq2 counts normalized on gene size in seven *Streptomyces* species of interest in trophophase and idiophase

Supplementary Table S7: Remarkable species regarding core gene order compared to the *Streptomyces* consensus

Supplementary Table S8: Annotation of *rrn* genes in *Streptomyces* from the panel

Supplementary Table S9: *rrn* operons features of interest to reproduce the analysis

Supplemental File 1: Script used to conduct data analyses

Supplemental File 2: *Streptomyces coelicolor* A3(2) complete GO annotation (GMT file)
