# Supplementary material for: Ribosomal RNA operons define a central functional compartment in the Streptomyces chromosome
Source: Nucleic Acids Res. 2022 Nov 21;50(20):11654–69. doi: 10.1093/nar/gkac1076 (PMC9723626; doi:10.1093/nar/gkac1076)
Supplement: gkac1076_Supplemental_Files [file gkac1076_supplemental_files.zip › Supplementary_Figure_S1_S3_S4_S6to9.pdf]

Ribosomal RNA operons define a central functional compartment in the *Streptomyces* chromosome

Jean-Noël Lorenzi[1], Annabelle Thibessard[2], Virginia S. Lioy[1], Frédéric Boccard[1], Pierre Leblond[2], Jean-Luc Pernodet[1], Stéphanie Bury-Moné[1],*

[1]Université Paris-Saclay, CEA, CNRS, Institute for Integrative Biology of the Cell (I2BC), 91198, Gif-sur-Yvette, France
[2]Université de Lorraine, INRAE, DynAMic, F-54000 Nancy, France
* To whom correspondence should be addressed. stephanie.bury-mone@i2bc.paris-saclay.fr

Present Address: Jean-Noël Lorenzi, CNRS UMR7592, Institut Jacques Monod, Université Paris Diderot, Paris, France ; Collège de France, CNRS, INSERM, PSL Research University, Paris, France

Supplementary Figures (except Supplementary Figures S2 and S5 which are available in separate files of 15 pages)

Supplementary Figure S1: Proposed *rrn* nomenclature based on core gene environments

Supplementary Figure S3: Pairwise comparison of the core genomes that are proposed to share rearrangements that occurred in their common ancestor (3 pages)

Supplementary Figure S4: Generalized linear model of the core region size based on the knowledge of the *rrn* operons (number,locations), the chromosome size and the *oriC* position

Supplementary Figure S6: Mean gene persistence in the regions surrounding the *rrn* operons

Supplementary Figure S7: GO enrichment analysis of the core genes which are mainly located in the central compartment (A) or in the terminal compartments (B)

Supplementary Figure S8: Level of gene persistence and expression along the chromosome of *Streptomyces* species of interest

Supplementary Figure S9: Level of gene expression over growth depending on gene category (core, non-core or SMBGCs) and location inside or outside the central compartment

A

rrn environment category	*rrn* environment id	Core gene -1§	Core gene closest to the *rrn* operon start position§	Core gene +1§	Number of genomes
Aa	**A**	**Orth_2229 / SCO1389 / SAM23877_1447**	**Orth_2335 / SCO1390 / SAM23877_1448**	**Orth_2240 / SCO1391 / SAM23877_1449**	**125**
	a1	Orth_3457 / SCO1388 / SAM23877_1446	Orth_2229	Orth_2335	1
	a2	Orth_3494 / SCO1152 / SAM23877_1235	Orth_2335	Orth_2240	1
Bb	**B**	**Orth_420 / SCO1789 / SAM23877_1854**	**Orth_9302 / SCO1796 / SAM23877_1863**	**Orth_5066 / SCO1797 / SAM23877_1864**	**124**
	b1	Orth_8822 / SCO1788 / SAM23877_1853	Orth_420	Orth_9302	3
Cc	**C**	**Orth_3539 / SCO3013 / SAM23877_3051**	**Orth_9623 / SCO3024 / SAM23877_3064**	**Orth_6428 / SCO3025 / SAM23877_3065**	**121**
	c1	Orth_8414 / SCO3011 / SAM23877_3049	Orth_3539	Orth_9623	4
	c2	Orth_4341 / SCO3172 / SAM23877_3221	Orth_9623	Orth_6428	1
	c3	Orth_9233 / SCO2966 / SAM23877_3001	Orth_9623	Orth_6428	1
Dd	**D**	**Orth_4139 / SCO4127 / SAM23877_3557**	**Orth_6483 / SCO4122 / SAM23877_3565**	**Orth_2534 / SCO4121 / SAM23877_3566**	**112**
	d1	Orth_7996 / SCO4128 / SAM23877_3556	Orth_4139	Orth_6483	10
	d2	Orth_6541 / SCO4137 / SAM23877_3546	Orth_7996	Orth_4139	1
	d3	Orth_7996	Orth_4139	Orth_1153 / SCO3615 / SAM23877_4004	1
Ee	**E**	**Orth_8528 / SCO3345 / SAM23877_4216**	**Orth_2277 / SCO3337 / SAM23877_4223**	**Orth_6749 / SCO3330 / SAM23877_4229**	**111**
	e1	Orth_8528	Orth_2277	Orth_8774 / SCO4366 / SAM23877_3359	1
	e2	Orth_2277	Orth_6749	Orth_4487 / SCO3323 / SAM23877_4236	4
[d/e] recombinants	[d/e1]	Orth_4139	Orth_2277	Orth_8528	12
	[d/e2]	Orth_6483	Orth_2277	Orth_6749	2
	[d/e3]	Orth_4139	Orth_8528	Orth_4097 / SCO3347 / SAM23877_4214	3
	[d/e4]	Orth_2277	Orth_6483	Orth_2534	3
	[d/e5]	Orth_6749	Orth_6483	Orth_2534	12
	[d/e6]	Orth_7996	Orth_4139	Orth_2277	1
Ff	**F**	**Orth_5289 / SCO5740 / SAM23877_5442**	**Orth_6126 / SCO5744 / SAM23877_5446**	**Orth_7379 / SCO5747 / SAM23877_5451**	**126**
	f1	Orth_6126	Orth_7379	Orth_6249 / SCO5749 / SAM23877_5453	2
Atypical/ ectopic rrn environments	g1	Orth_2159 / SCO2538 / SAM23877_2587	Orth_2308 / SCO2533 / SAM23877_2584	Orth_3295 / SCO2547 / SAM23877_2592	1
	h1	Orth_1360 / SCO4979 / SAM23877_4794	Orth_5738 / SCO5005 / SAM23877_4811	Orth_7524 / SCO5009 / SAM23877_4815	2
	h2	Orth_7601 / SCO4975 / SAM23877_4786	Orth_1360	Orth_5738	1
	i1	Orth_5509 / SCO5291 / SAM23877_5067	Orth_9438 / SCO5353 / SAM23877_5097	Orth_7899 / SCO5354 / SAM23877_5098	1
	j1	Orth_75 / SCO5900 / SAM23877_5578	Orth_9758 / SCO5901 / SAM23877_5579	Orth_2449 / SCO5998 / SAM23877_5714	6
	j2	Orth_9758	Orth_2449	Orth_506 / SCO5999 / SAM23877_5716	1
	k1	Orth_4148 / SCO6060 / SAM23877_5773	Orth_719 / SCO6061 / SAM23877_5774	Orth_1203 / SCO6083 / SAM23877_5801	4

B

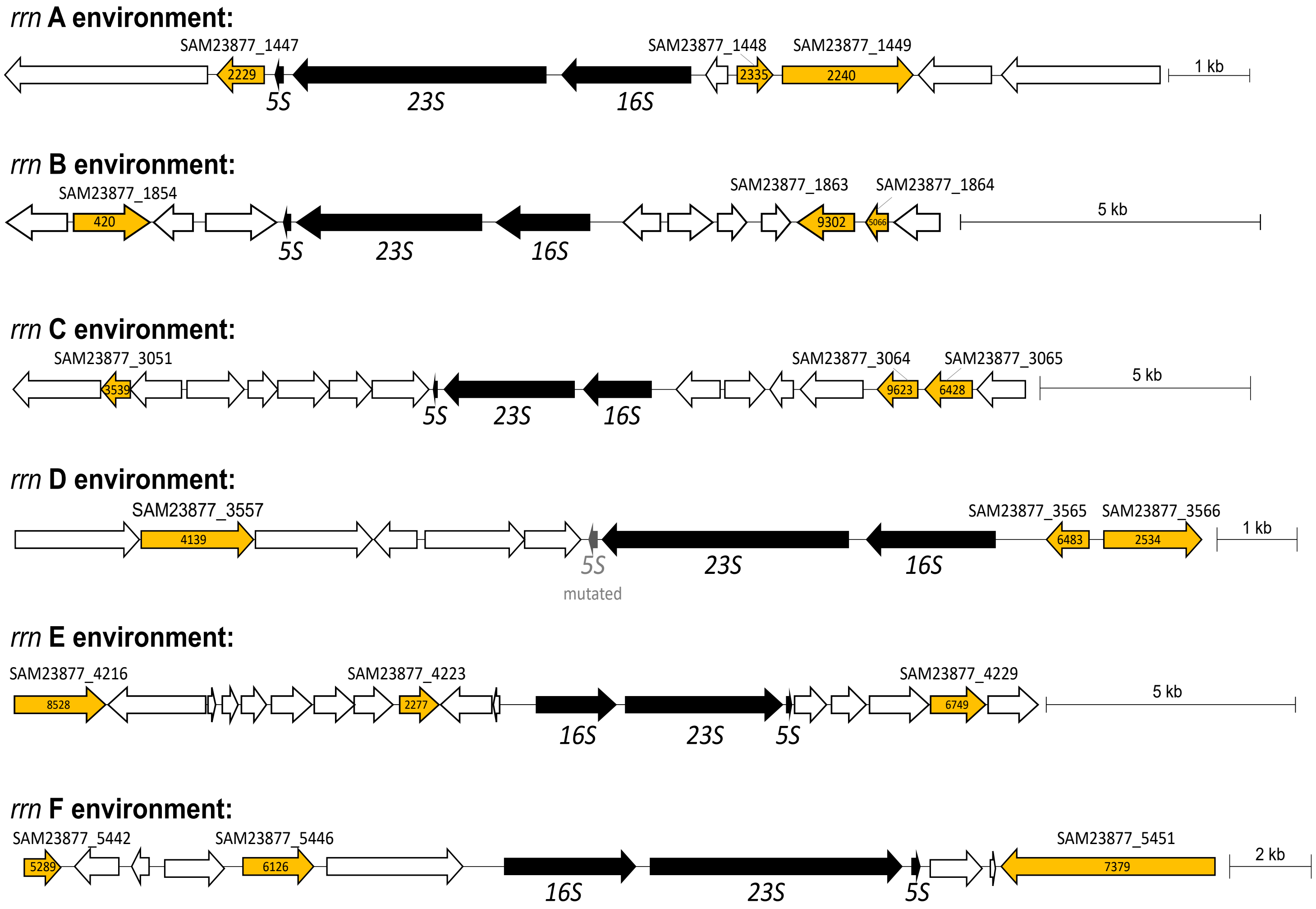

Figure S1: Proposed *rrn* nomenclature based on core gene environments

A. **Core gene environments characterized around all the *rrn* of the panel.** Canonical *rrn* neighborhoods are in bold. When first cited, the core gene identifiers are indicated as follows: ortholog group number ('Orth_#') based on the re-annotation performed in this study / *S. coelicolor* ('SCO#') Uniprot identifier / *S. ambofaciens* ATCC 23877 ('SAM23877_#') Uniprot identifier. If already cited, the CDS is then identified solely on the basis of its ortholog group number. These two strains were chosen for reference because *S. coelicolor* A3(2) corresponds to the most documented *Streptomyces* strain, and *S. ambofaciens* ATCC 23877 is another model *Streptomyces*, closest to the ancestral consensus in terms of core gene order. 'Core gene -1' and 'Core gene +1' designate the core genes located upstream and downstream of the core gene closest to the beginning of the *rrn* operon, respectively. The *rrn* environments are oriented according to the consensus order as found in *S. viridosporus* T7A ATCC 39115. An asterisk is used in the nomenclature to indicate that the motif is in the opposite orientation to that shown above.

B. **Schematic representation of the canonical *rrn* environments in *S. ambofaciens* ATCC 23877.** Core genes that are a signature of the *rrn* environment are in orange. The numbers in each arrow correspond to the annotation of the orthologous groups performed in this study (annotation of all genomes in the same manner is available in **Supplementary Table S2**).

Figure S3 (1/3)

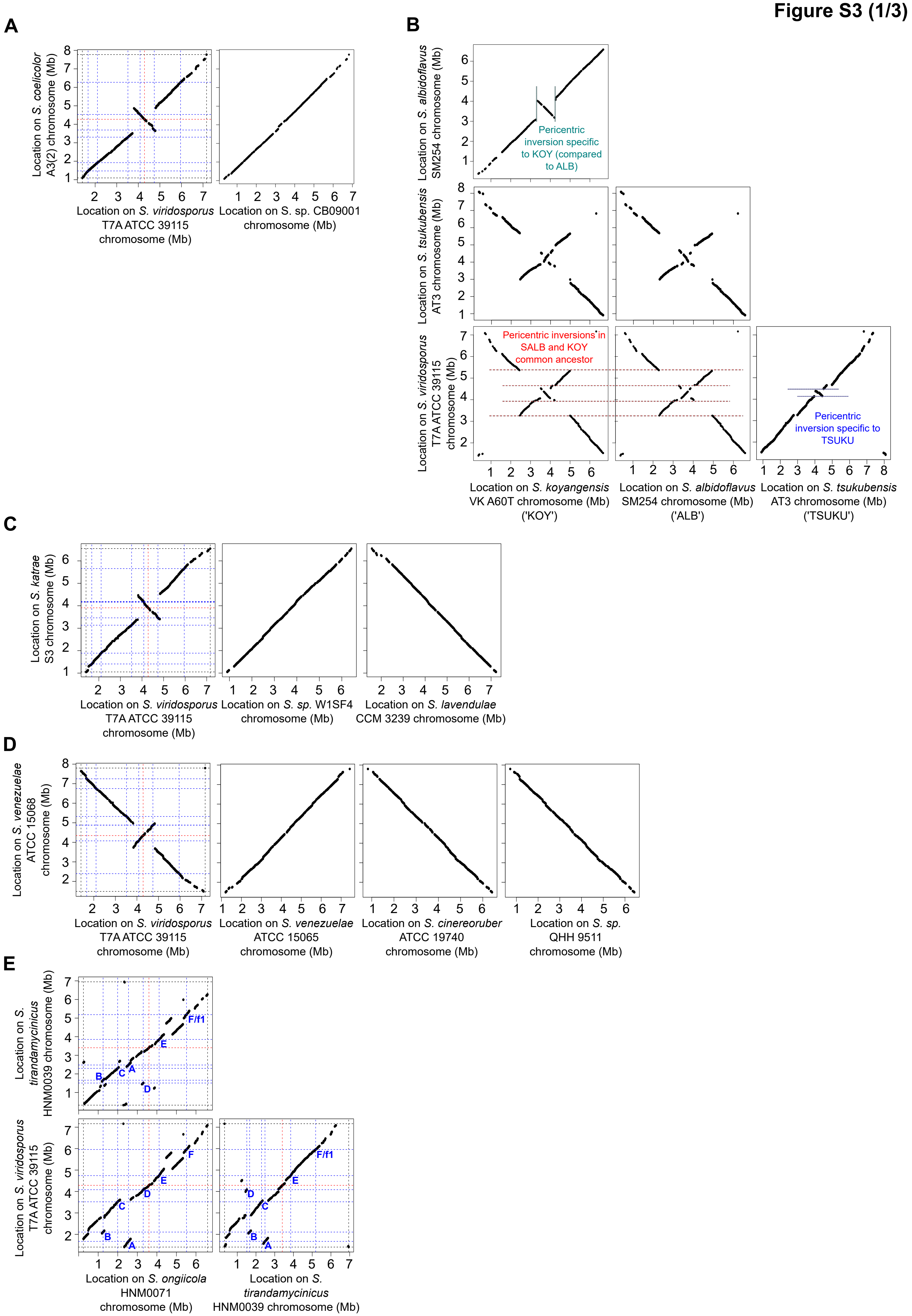

A
Location on S. coelicolor A3(2) chromosome (Mb)
Location on S. viridosporus T7A ATCC 39115 chromosome (Mb)
Location on S. sp. CB09001 chromosome (Mb)
B
Location on S. albidoflavus SM254 chromosome (Mb)
Pericentric inversion specific to KOY (compared to ALB)
Location on S. tsukubensis AT3 chromosome (Mb)
Location on S. viridosporus T7A ATCC 39115 chromosome (Mb)
Pericentric inversions in SALB and KOY common ancestor
Pericentric inversion specific to TSUKU
Location on S. koyangensis VK A60T chromosome (Mb) ('KOY')
Location on S. albidoflavus SM254 chromosome (Mb) ('ALB')
Location on S. tsukubensis AT3 chromosome (Mb) ('TSUKU')
C
Location on S. katrae S3 chromosome (Mb)
Location on S. viridosporus T7A ATCC 39115 chromosome (Mb)
Location on S. sp. W1SF4 chromosome (Mb)
Location on S. lavendulae CCM 3239 chromosome (Mb)
D
Location on S. venezuelae ATCC 15068 chromosome (Mb)
Location on S. viridosporus T7A ATCC 39115 chromosome (Mb)
Location on S. venezuelae ATCC 15065 chromosome (Mb)
Location on S. cinereoruber ATCC 19740 chromosome (Mb)
Location on S. sp. QHH 9511 chromosome (Mb)
E
Location on S. tirandamycinicus HNM0039 chromosome (Mb)
F/f1
E
A
B
C
D
Location on S. viridosporus T7A ATCC 39115 chromosome (Mb)
F
Location on S. ongiicola HNM0071 chromosome (Mb)
Location on S. tirandamycinicus HNM0039 chromosome (Mb)

Figure S3 (2/3)

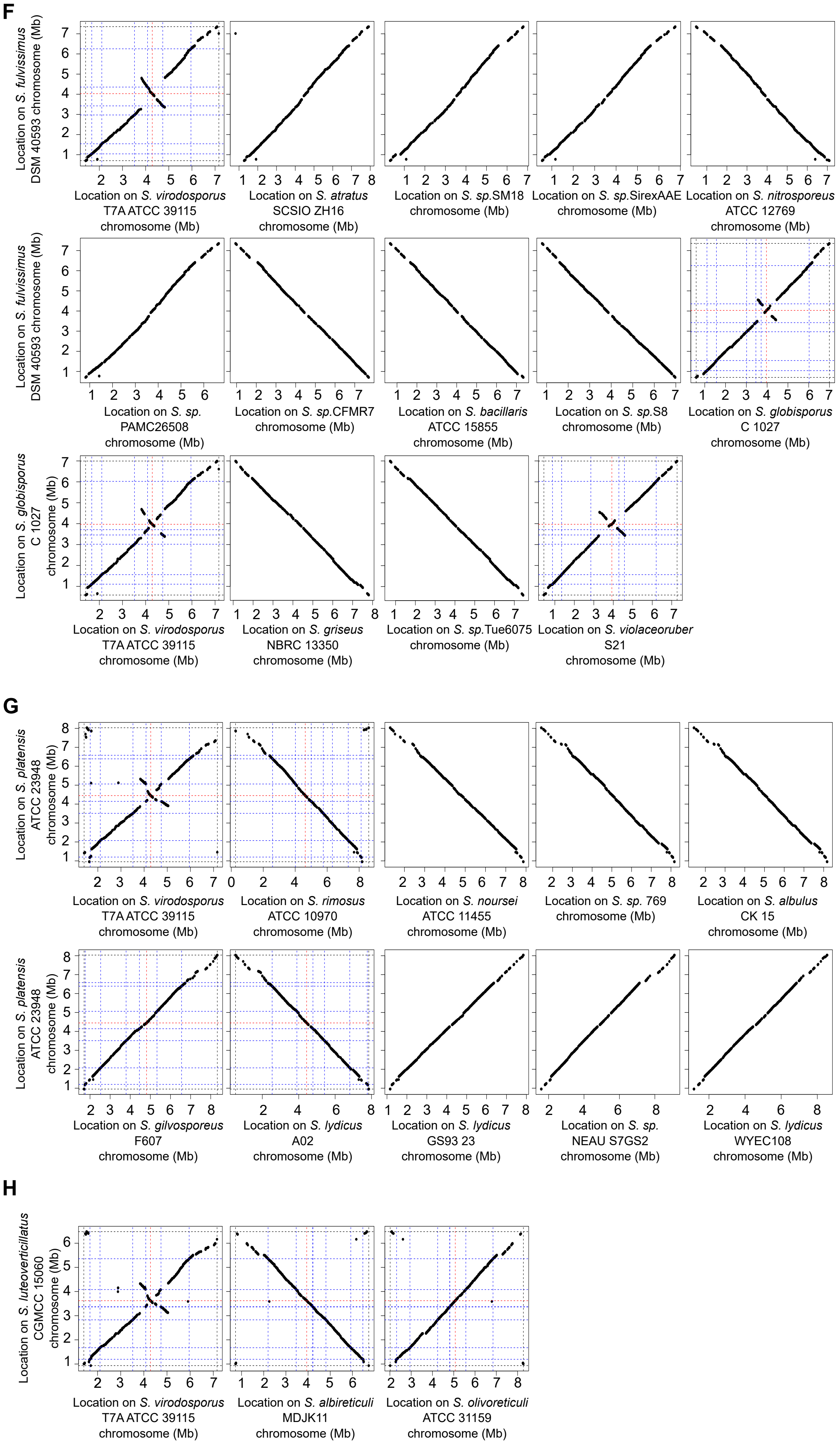
F
Location on S. fulvissimus DSM 40593 chromosome (Mb)
Location on S. virodosporus T7A ATCC 39115 chromosome (Mb)
Location on S. atratus SCSIO ZH16 chromosome (Mb)
Location on S. sp.SM18 chromosome (Mb)
Location on S. sp.SirexAAE chromosome (Mb)
Location on S. nitrosporeus ATCC 12769 chromosome (Mb)
Location on S. fulvissimus DSM 40593 chromosome (Mb)
Location on S. sp. PAMC26508 chromosome (Mb)
Location on S. sp.CFMR7 chromosome (Mb)
Location on S. bacillaris ATCC 15855 chromosome (Mb)
Location on S. sp.S8 chromosome (Mb)
Location on S. globisporus C 1027 chromosome (Mb)
Location on S. globisporus C 1027 chromosome (Mb)
Location on S. virodosporus T7A ATCC 39115 chromosome (Mb)
Location on S. griseus NBRC 13350 chromosome (Mb)
Location on S. sp.Tue6075 chromosome (Mb)
Location on S. violaceoruber S21 chromosome (Mb)
G
Location on S. platensis ATCC 23948 chromosome (Mb)
Location on S. virodosporus T7A ATCC 39115 chromosome (Mb)
Location on S. rimosus ATCC 10970 chromosome (Mb)
Location on S. noursei ATCC 11455 chromosome (Mb)
Location on S. sp. 769 chromosome (Mb)
Location on S. albulus CK 15 chromosome (Mb)
Location on S. platensis ATCC 23948 chromosome (Mb)
Location on S. gilvosporeus F607 chromosome (Mb)
Location on S. lydicus A02 chromosome (Mb)
Location on S. lydicus GS93 23 chromosome (Mb)
Location on S. sp. NEAU S7GS2 chromosome (Mb)
Location on S. lydicus WYEC108 chromosome (Mb)
H
Location on S. luteoverticillatus CGMCC 15060 chromosome (Mb)
Location on S. virodosporus T7A ATCC 39115 chromosome (Mb)
Location on S. albireticuli MDJK11 chromosome (Mb)
Location on S. olivoreticuli ATCC 31159 chromosome (Mb)

I

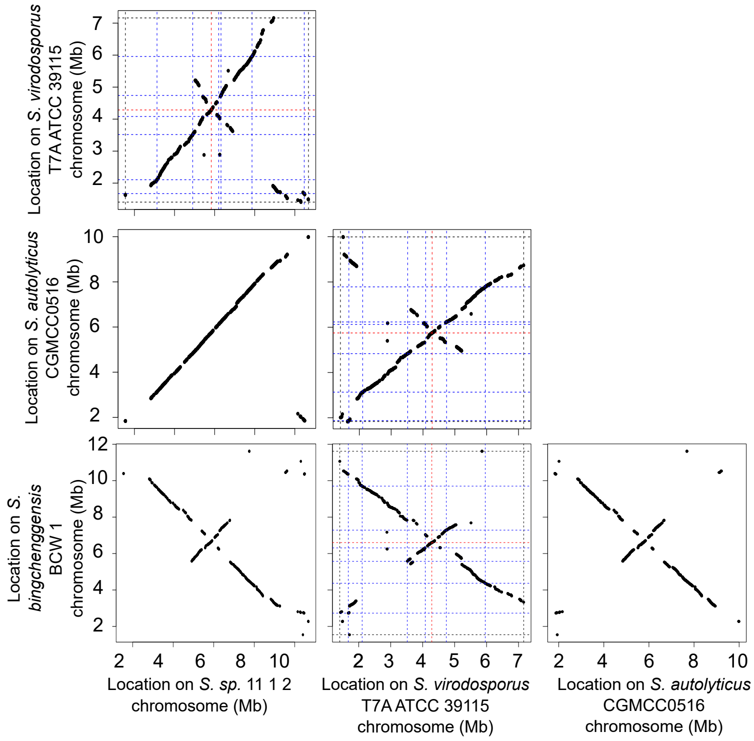

Figure S3: Pairwise comparison of the core genomes that are proposed to share rearrangements that occurred in their common ancestor

Panels A to I present the pairwise comparisons that support the occurrence of intra-chromosomal rearrangement in the ancestor of strains of interest. In each case, a comparison to the consensus strain (*S. viridosporus* T7A ATCC 39115) is added as a reference, as well as the indication of the positioning of *rrn* operons (blue), the origin of replication (red) and the core genome ends (black) for at least one pairwise comparison. In the case of very complex rearrangements (panel E), the name of the *rrn* according to the nomenclature proposed in this study has been indicated. The presence of an unbroken diagonal indicates that the strains have identically ordered core genomes.

A

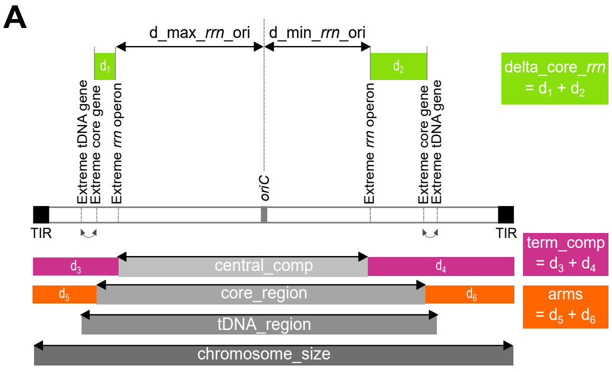

B

$$y_i = \beta_0 + \beta_1.x_{i1} + \beta_2.x_{i2} + \beta_3.x_{i3} + \beta_4.x_{i4} + \varepsilon_i$$

where y_i is the core region size (in bp) of the ith genome

	Estimate	Standard error	*t* value	Pr(>|t|)	
Intercept	β_0 = 1.016e+06	4.974e+05	2.042	0.043332	*
Maximal distance between *rrn* and *oriC* (x_{i1})	β_1 = 7.262e-01	2.603e-01	2.790	0.006136	**
Chromosome size (x_{i2})	β_2 = 2.398e-01	3.970e-02	6.041	1.77e-08	***
Central compartment size (x_{i3})	β_3 = 5.522e-01	1.468e-01	3.762	0.000262	***
Number of *rrn* genes (x_{i4})	β_3 = -6.694e+04	2.036e+04	-3.288	0.001323	**

	Min	First quartile	Median	Third quartile	Max
Residuals (ε_i)	-892177	-174499	-22613	164547	1663768

Residual standard error: 312800 bp on 120 degrees of freedom
Multiple R^2: 0.8639, Adjusted R^2: 0.8594
F-statistic: 190.4 on 4 and 120 degrees of freedom, *p*-value: < 2.2e-16
AIC: 3524.939, 6 degrees of freedom

C

	Degrees of freedom	Sum of Squares	Mean square	F value	Pr(>F)	
Maximal distance between *rrn* and *oriC* (x_{i1})	1	6.9333e+13	6.9333e+13	708.736	< 2.2e-16	***
Chromosome size (x_{i2})	1	2.9697e+12	2.9697e+12	30.357	2.086e-07	***
Central compartment size (x_{i3})	1	1.1600e+12	1.1600e+12	11.858	0.0007914	***
Number of *rrn* genes (x_{i4})	1	1.0578e+12	1.0578e+12	10.813	0.0013229	**
Residuals (ε_i)	120	1.1739e+13	9.7826e+10			

D

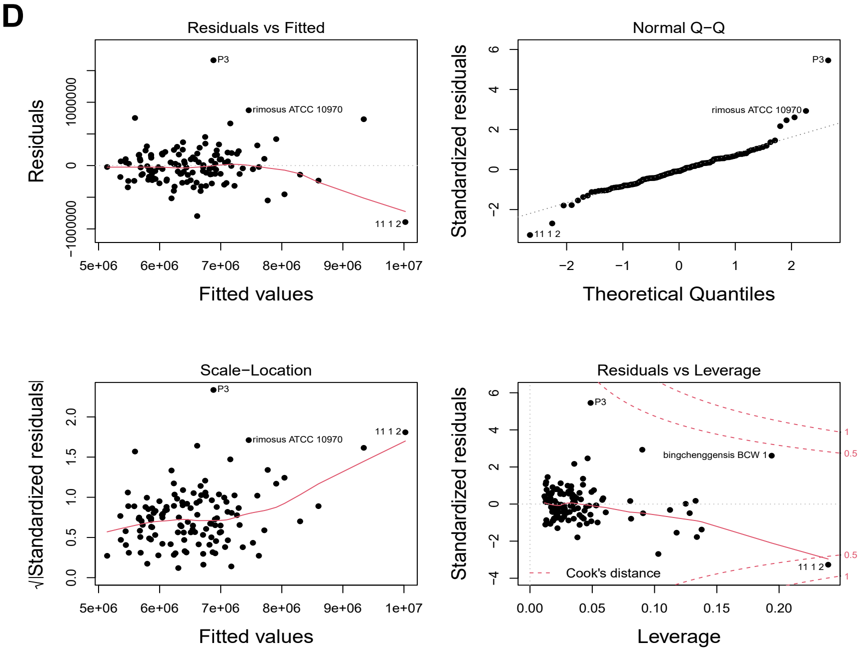

Figure S4: Generalized linear model of the core region size based on the knowledge of the *rrn* operons (number,locations), the chromosome size and the *oriC* position

A. Schematic representation of the *Streptomyces* regions and distances analyzed in the study. The *rrn* genes are always contained in the core region, while the relative positions of the extreme tDNA and core genes may differ from genome to genome (being thus different from those represented in this panel). The location of these latter is therefore represented by bijective grey arrows. The cumulative distance between the extreme *rrn* and core genes was further termed ‘delta_core_*rrn*’. The maximal and minimal distances of *oriC* to the most extreme *rrn* (‘d_max_*rrn*_ori’ and ‘d_min_*rrn*_ori’) were also determined. Other abbreviations: ‘term_comp’ = terminal compartments (cumulative size of the left and right compartments), TIR = terminal inverted repeats.

B. Summary of the ANOVA model based on the sizes of the central compartment and the chromosome, the maximal distance of *rrn* genes to the origin of replication and the number of *rrn* genes. The explanatory variables are ranked according to their selection order in a forward regression approach. Code: ‘***’ (p< 0.001), ‘**’ (p< 0.01), ‘*’ (p< 0.05), ‘.’ (p< 0.1). AIC = Akaike Information Criterion

C. Type I analysis of variance (ANOVA) sum test on the model. In this type of test, the *p*-value of each explanatory variable depends on its order in the model. Code: ‘***’ (p< 0.001), ‘**’ (p< 0.01), ‘*’ (p< 0.05), ‘.’ (p< 0.1).

D. Diagnostic scatterplots evaluating the assumptions of linearity, homoscedasticity and bivariate normality as well as the leverage effect. The plot of the residuals *versus* fitted values addresses both the linearity and homoscedasticity (constant variability) assumptions: If the relationship is linear, the red line in the center of the graph is fairly flat, without distinct patterns; in case of homoscedasticity, the error is relatively constant across all of the fitted values. The assumption of bivariate normality is taken into account with a Quantile-Quantile (Q-Q) normal graph: In case of bivariate normality, the points should follow the straight dashed red line. The scale-location plot also diagnoses the homoscedasticity, a horizontal line with equally spread points being a good indicator. The last graph evaluates the ‘leverage effect’ i.e. allow to identify genomes that may change the regression line. Points presenting a Cook’s distance greater than 1 should be excluded from the model (no case in this study). Based on these analyses, genomes that show a rather unusual pattern are indicated on each graph.

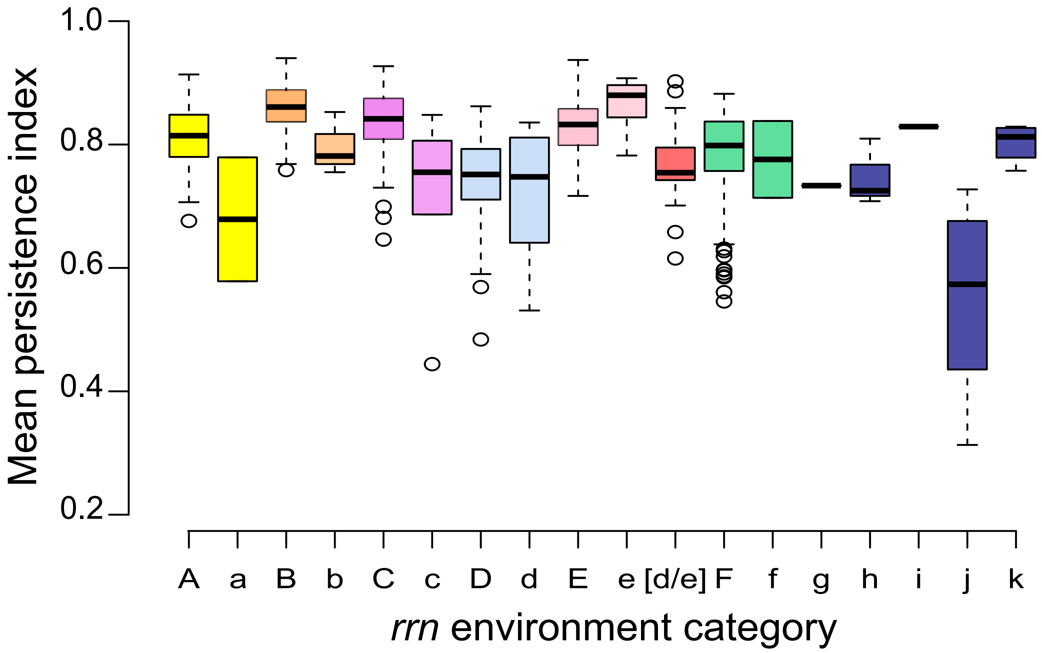

Figure S6: Mean gene persistence in the regions surrounding the *rrn* operons

The mean persistence was calculated within a window of 81 CDS centered on the *rrn* operon of interest. The boxplot represents the first quartile, median and third quartile. The upper whisker extends from the hinge to the largest value no further than 1.5 * the inter-quartile range (IQR, i.e. distance between the first and third quartiles) from the hinge. The lower whisker extends from the hinge to the smallest value at most 1.5 * IQR of the hinge. Outliers are represented (dots). The number of *rrn* operons in each category is presented in **Figure 1.C**.

A

Processes		Adjusted *p* value	#
metabolic process	GO:0008152	1,6E-36	425
organonitrogen compound metabolic process	GO:1901564	7,4E-32	243
biosynthetic process	GO:0009058	1,2E-31	254
organic substance metabolic process	GO:0071704	2,5E-30	368
cellular metabolic process	GO:0044237	2,2E-29	281
organonitrogen compound biosynthetic process	GO:1901566	4,4E-26	123
cellular biosynthetic process	GO:0044249	2,2E-24	207
carboxylic acid metabolic process	GO:0019752	1,1E-23	106
primary metabolic process	GO:0044238	5,6E-21	275
cellular amino acid metabolic process	GO:0006520	8,7E-21	84
nitrogen compound metabolic process	GO:0006807	1,2E-20	171
cellular process	GO:0009987	2,5E-20	363
organic substance biosynthetic process	GO:1901576	7,2E-20	160
oxoacid metabolic process	GO:0043436	1,2E-16	89
cellular amino acid biosynthetic process	GO:0008652	4,1E-16	59
small molecule metabolic process	GO:0044281	1,6E-15	111
purine nucleotide metabolic process	GO:0006163	7,5E-15	35
purine nucleotide biosynthetic process	GO:0006164	8,5E-15	33
alpha-amino acid metabolic process	GO:1901605	7,8E-14	59
purine ribonucleotide biosynthetic process	GO:0009152	4,8E-13	30
metal ion binding	GO:0046872	1,9E-12	159
cation binding	GO:0043169	3,5E-11	152
alpha-amino acid biosynthetic process	GO:1901607	4,5E-11	41
rRNA binding	GO:0019843	7,3E-11	31
cytosolic large ribosomal subunit	GO:0022625	1,2E-10	17
nucleotide binding	GO:0000166	1,6E-10	169
nucleotide biosynthetic process	GO:0009165	1,9E-10	32
macromolecule metabolic process	GO:0043170	2,4E-10	178
nucleotide metabolic process	GO:0009117	3,3E-10	38
organic acid biosynthetic process	GO:0016053	4,9E-10	45
ligase activity	GO:0016874	3,0E-09	54
RNA binding	GO:0003723	3,2E-09	70
catalytic activity, acting on RNA	GO:0140098	5,7E-09	38
catalytic activity	GO:0003824	1,0E-08	484
small ribosomal subunit	GO:0015935	1,8E-08	16
translation	GO:0006412	2,0E-08	68
ribosome	GO:0005840	3,4E-08	16
tRNA binding	GO:0000049	3,7E-08	19
cellular macromolecule biosynthetic process	GO:0034645	5,5E-08	97
peptide biosynthetic process	GO:0043043	7,6E-08	67
magnesium ion binding	GO:0000287	8,3E-08	42
cellular aromatic compound metabolic process	GO:0006725	1,0E-07	79
cytosolic small ribosomal subunit	GO:0022627	1,1E-07	11
large ribosomal subunit	GO:0015934	1,3E-07	17
organic acid metabolic process	GO:0006082	1,6E-07	56
organic cyclic compound biosynthetic process	GO:1901362	1,6E-07	74
sulfur compound metabolic process	GO:0006790	2,0E-07	32
protein metabolic process	GO:0019538	2,9E-07	106
ribonucleoprotein complex	GO:1990904	3,4E-07	18
ATP metabolic process	GO:0046034	1,7E-06	18
tRNA metabolic process	GO:0006399	1,7E-06	23
tRNA aminoacylation	GO:0043039	2,4E-06	18
nucleoside phosphate metabolic process	GO:0006753	3,9E-06	29
ATP binding	GO:0005524	4,7E-06	126
ribonucleoside monophosphate biosynthetic process	GO:0009156	4,8E-06	16
cellular protein metabolic process	GO:0044267	8,1E-06	90
nucleobase-containing compound metabolic process	GO:0006139	8,2E-06	72
NADH dehydrogenase activity	GO:0003954	8,7E-06	17
ncRNA processing	GO:0034470	1,2E-05	22
adenyl ribonucleotide binding	GO:0032559	1,3E-05	126

Processes		Adjusted *p* value	#
lyase activity	GO:0016829	3,2E-05	47
aerobic respiration	GO:0009060	3,8E-05	21
quinone binding	GO:0048038	5,6E-05	15
tRNA aminoacylation for protein translation	GO:0006418	6,1E-05	16
RNA processing	GO:0006396	7,4E-05	20
nucleoside-triphosphatase activity	GO:0017111	7,6E-05	31
generation of precursor metabolites and energy	GO:0006091	7,7E-05	28
aminoacyl-tRNA ligase activity	GO:0004812	1,1E-04	16
IMP metabolic process	GO:0046040	1,2E-04	12
pyrophosphatase activity	GO:0016462	1,7E-04	29
oxidoreductase activity, acting on NAD(P)H	GO:0016651	1,8E-04	19
oxidoreduction-driven active transmembrane transporter activity	GO:0015453	1,9E-04	17
branched-chain amino acid metabolic process	GO:0009081	2,0E-04	14
purine ribonucleoside monophosphate biosynthetic process	GO:0009168	2,1E-04	15
transferase activity	GO:0016740	2,2E-04	158
hydrolase activity, acting on acid anhydrides, in phosphorus-containing anhydrides	GO:0016818	2,2E-04	30
RNA modification	GO:0009451	2,3E-04	19
phosphorus metabolic process	GO:0006793	2,8E-04	57
ribosome biogenesis	GO:0042254	3,6E-04	16
electron transfer activity	GO:0009055	4,4E-04	24
cell cycle	GO:0007049	9,1E-04	15
NADH dehydrogenase (ubiquinone) activity	GO:0008137	1,0E-03	13
histidine biosynthetic process	GO:0000105	1,2E-03	10
electron transport chain	GO:0022900	1,6E-03	23
histidine metabolic process	GO:0006547	1,6E-03	11
NADH dehydrogenase (quinone) activity	GO:0050136	2,3E-03	14
ATP synthesis coupled proton transport	GO:0015986	2,3E-03	8
proton-transporting ATP synthase activity, rotational mechanism	GO:0046933	2,3E-03	8
branched-chain amino acid biosynthetic process	GO:0009082	2,5E-03	12
pyruvate metabolic process	GO:0006090	2,6E-03	16
'de novo' IMP biosynthetic process	GO:0006189	2,8E-03	10
proton-transporting ATP synthase complex	GO:0045259	4,1E-03	8
tRNA modification	GO:0006400	4,7E-03	12
proton transmembrane transport	GO:1902600	4,7E-03	17
cellular respiration	GO:0045333	5,0E-03	16
tRNA processing	GO:0008033	6,2E-03	14
cellular amino acid catabolic process	GO:0009063	6,9E-03	17
phosphate ion transport	GO:0006817	7,0E-03	8
cell division	GO:0051301	7,5E-03	18
transition metal ion binding	GO:0046914	8,5E-03	57
ATP hydrolysis activity	GO:0016887	8,6E-03	22
ATP biosynthetic process	GO:0006754	1,1E-02	8
ligase activity, forming carbon-nitrogen bonds	GO:0016879	1,6E-02	21
channel activity	GO:0015267	1,7E-02	10
oxidative phosphorylation	GO:0006119	1,7E-02	10
NAD(P)H dehydrogenase (quinone) activity	GO:0003955	1,7E-02	10
iron-sulfur cluster assembly	GO:0016226	1,7E-02	7
pentosyltransferase activity	GO:0016763	1,8E-02	12
rRNA processing	GO:0006364	1,8E-02	12
tryptophan metabolic process	GO:0006568	2,2E-02	9
ATP synthesis coupled electron transport	GO:0042773	2,7E-02	8
oxidoreductase activity, acting on NAD(P)H, quinone or similar compound as acceptor	GO:0016655	2,8E-02	11
sulfur amino acid metabolic process	GO:0000096	3,7E-02	13
protein catabolic process	GO:0030163	4,1E-02	10
glycolytic process	GO:0006096	4,1E-02	10
phosphate ion transmembrane transport	GO:0035435	4,1E-02	5
folic acid-containing compound metabolic process	GO:0006760	4,3E-02	9
hydro-lyase activity	GO:0016836	4,3E-02	18
respiratory electron transport chain	GO:0022904	4,6E-02	11
zinc ion binding	GO:0008270	4,7E-02	35

B

Processes		Adjusted *p* value	#
phospholipid biosynthetic process	GO:0008654	7,7E-04	7
phospholipid metabolic process	GO:0006644	1,9E-03	7
cellular lipid metabolic process	GO:0044255	2,5E-03	11
catalytic activity	GO:0003824	5,6E-03	70
glycerophospholipid biosynthetic process	GO:0046474	7,9E-03	4
glycerophospholipid metabolic process	GO:0006650	1,3E-02	4
nucleobase-containing compound metabolic process	GO:0006139	2,7E-02	15
lipid metabolic process	GO:0006629	3,0E-02	10
lipid biosynthetic process	GO:0008610	4,0E-02	9
organic substance metabolic process	GO:0071704	4,5E-02	45
base-excision repair	GO:0097510	4,9E-02	2

0 36

Negative log 10 of adjusted *p* values

Figure S7: GO enrichment analysis of the core genes which are mainly located in the central compartment (A) or in the terminal compartments (B)

The statistically enriched processes were identified by a g:Profiler analysis including 748 genes whose GO annotation was available. The symbol “#” indicates the number of these core genes in each GO category. Some genes belong to several GO categories. The same scale was used for both panels.

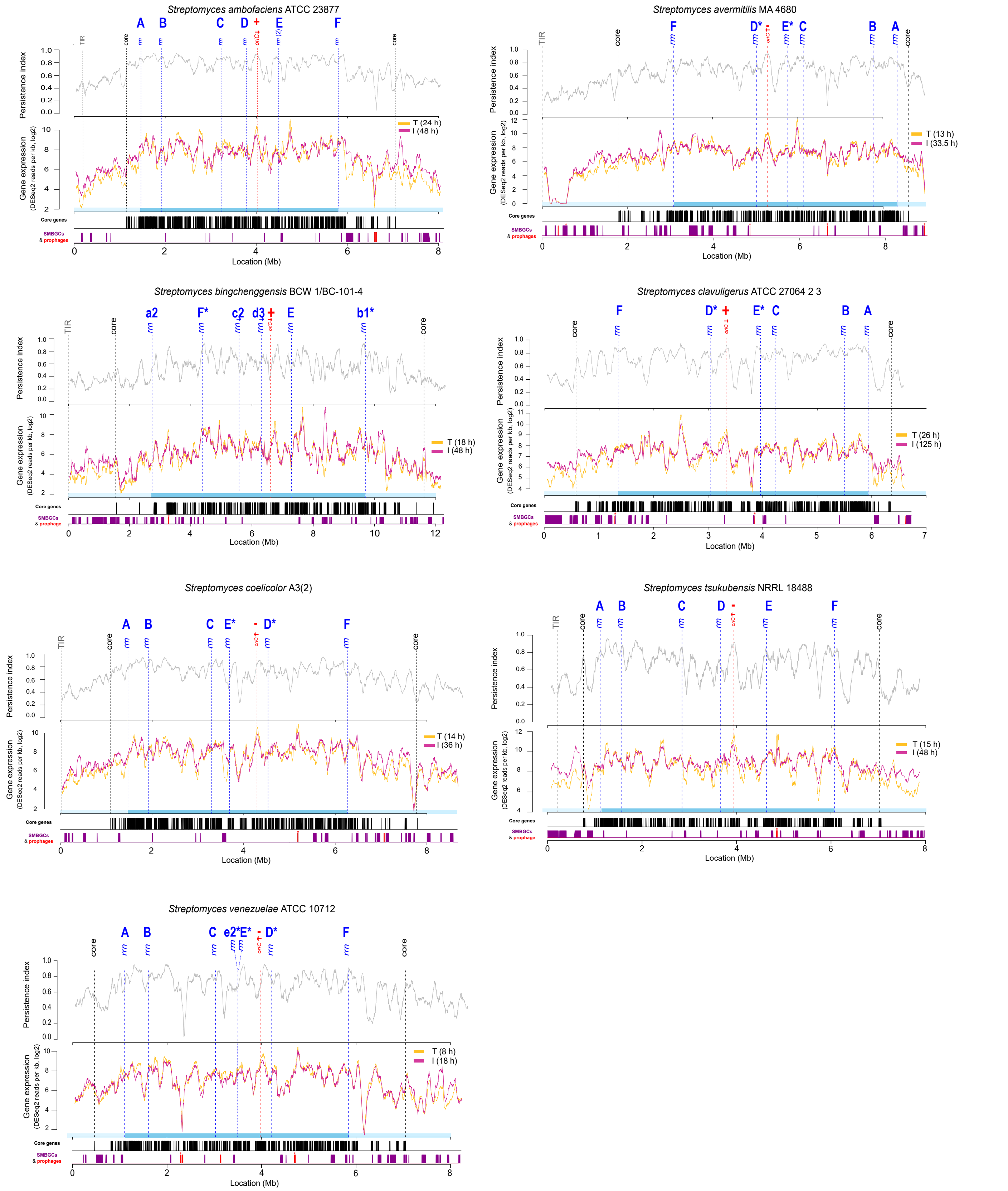

Figure S8: Level of gene persistence and expression along the chromosome of *Streptomyces* species of interest

The level of gene persistence (*y*-axis, top) and expression (*y*-axis, bottom) along the chromosome (*x*-axis) is represented using a sliding window [81 coding sequences (CDSs), with 1 CDS steps]. Gene expression corresponds to the DESeq2 normalized counts (log2) measured in cells harvested during the trophophase (T) and the idiophase (I), the time points being indicated for each species (see Methods section for further details). The positions of the most extreme core genes ('core', black lines) and of all *rrn* operon (blue lines) are indicated. The genomic sequences are in the orientation provided by the databases, but the nomenclature used to identify each *rrn* is in reference to an order of the consensus core genome corresponding to '*rrn* ABCDEF *dnaA*+'. The origin of replication (*oriC*) is defined regarding the position of the *dnaA* gene, the red arrow representing the orientation of this gene, and the sign ('+' or '-') its orientation compared to the canonical organization of the chromosome ('*rrn* ABCDEF *dnaA*+'). The '*rnn*(2)' label indicates the position of the unlinked operon (containing only 16S and 23S rRNAs) in *S. ambofaciens* ATCC 23877. Arrows under *rrn* operons in *S. bingchenggensis* BCW-1 chromosome indicate lagging orientations (relative to the direction of chromosome replication). The density of the core genes as well as the positions of SMBGCs (predicted by AntiSMASH5.0) and prophages (predicted by PHASTER) are indicated under the graph. Incomplete prophages are indicated by an asterisk. Abbreviation: TIR (terminal inverted repeat).

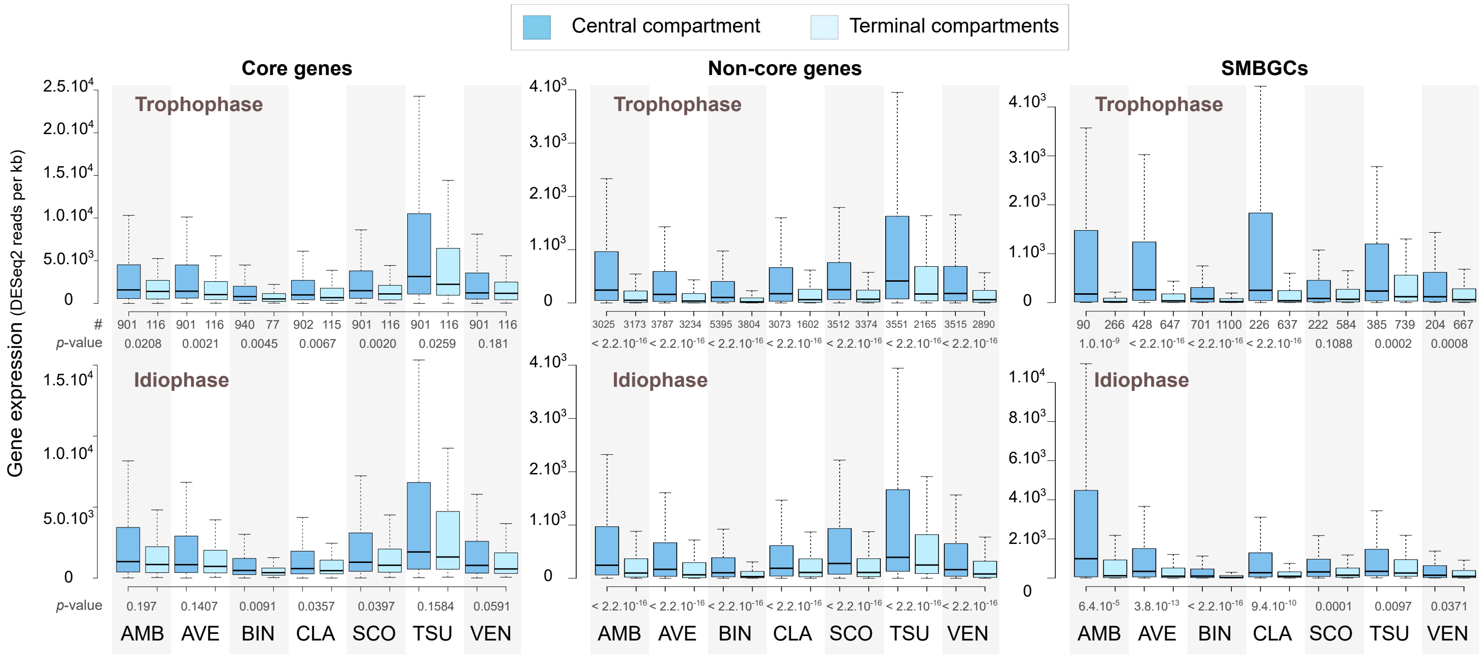

AMB	*S. ambofaciens* ATCC 23877
AVE	*S. avermitilis* MA 4680
BIN	*S. bingchenggensis* BCW 1
CLA	*S. clavuligerus* ATCC 27064 2 3
SCO	*S. coelicolor* A3(2)
TSU	*S. tsukubensis* NRRL 18488
VEN	*S. venezuelae* ATCC 10712

Figure S9: Level of gene expression over growth depending on gene category (core, non -core or SMBGCs) and location inside or outside the central compartment

The trophophase time points correspond to 24h, 13h, 18h, 26h, 14h, 15h, 8h for *S. ambofaciens* ATCC 23877[1] (AMB), *S. avermitilis* MA 4680[2] (AVE), S. *bingchenggensis* BCW 1/BC-101-4[3] (BIN), *S. clavuligerus* ATCC 27064 2 3[2] (CLA), *S. coelicolor* A3(2) (SCO)[4], *S. tsukubensis* NRRL 18488[2] (TSU) and *S. venezuelae* ATCC 10712[5] (VEN), respectively. The idiophase time points correspond to 48h, 33.5h, 48h, 125h, 36h, 48h, 18h for *S. ambofaciens* ATCC 23877[1], *S. avermitilis* MA 4680[2], S. *bingchenggensis* BCW 1/BC-101-4[3], *S. clavuligerus* ATCC 27064 2 3[2], *S. coelicolor* A3(2)[4], *S. tsukubensis* NRRL 18488[2] and *S. venezuelae* ATCC 10712[5], respectively. Gene transcription (in sense orientation) corresponds to the number of DESeq2 normalized reads per kb. The boxplot represents the first quartile, median and third quartile. The upper whisker extends from the hinge to the largest value no further than 1.5 * IQR from the hinge. The lower whisker extends from the hinge to the smallest value at most 1.5 * IQR of the hinge. For clarity, outliers were not represented. Under each bar, the number of genes (#) in each category is indicated as well as the *p*-value of two-sided Wilcoxon rank sum tests with continuity correction (comparing of gene expression in central *versus* terminal compartments).

Transcriptome references:

1. Lioy, V. S. *et al.* Dynamics of the compartmentalized *Streptomyces* chromosome during metabolic differentiation. *Nat Commun* **12**, 5221 (2021).
2. Kim, W. *et al.* Transcriptome and translatome profiles of *Streptomyces* species in different growth phases. *Sci Data* **7**, 138 (2020).
3. Jin, P. *et al.* Mining and fine-tuning sugar uptake system for titer improvement of milbemycins in *Streptomyces bingchenggensis*. *Synthetic and Systems Biotechnology* **5**, 214–221 (2020).
4. Jeong, Y. *et al.* The dynamic transcriptional and translational landscape of the model antibiotic producer *Streptomyces coelicolor* A3(2). *Nat Commun* **7**, 11605 (2016).
5. Gehrke, E. J. *et al.* Silencing cryptic specialized metabolism in *Streptomyces* by the nucleoid-associated protein Lsr2. *Elife* **8**, (2019).
