# Supplementary material for: Ribosomal RNA operons define a central functional compartment in the Streptomyces chromosome
Source: Nucleic Acids Res. 2022 Nov 21;50(20):11654–69. doi: 10.1093/nar/gkac1076 (PMC9723626; doi:10.1093/nar/gkac1076)
Supplement: gkac1076_Supplemental_Files [file gkac1076_supplemental_files.zip › Supplementary_Figure_S2.pdf]

Figure S2 (1/15)

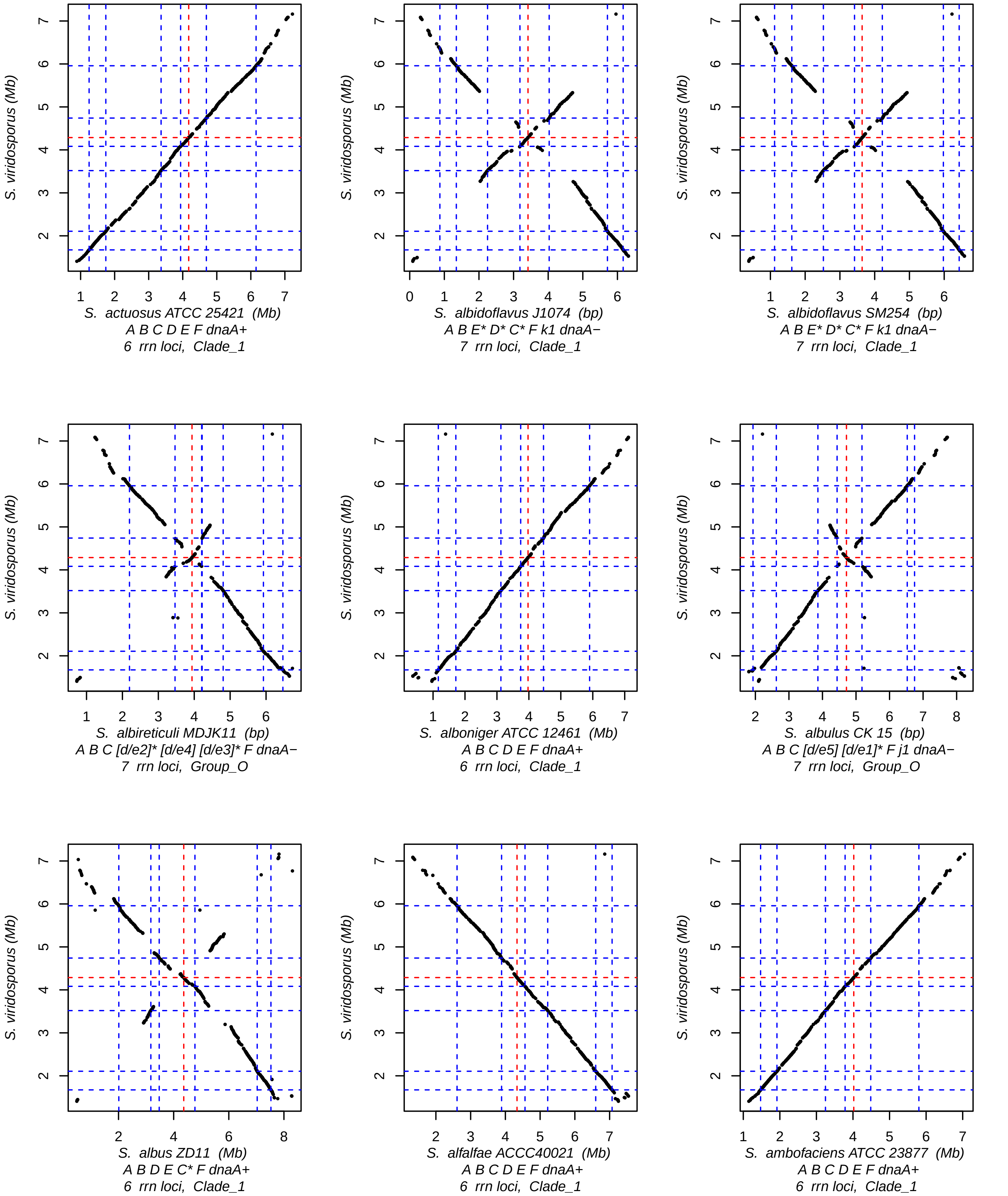

Figure S2 (2/15)

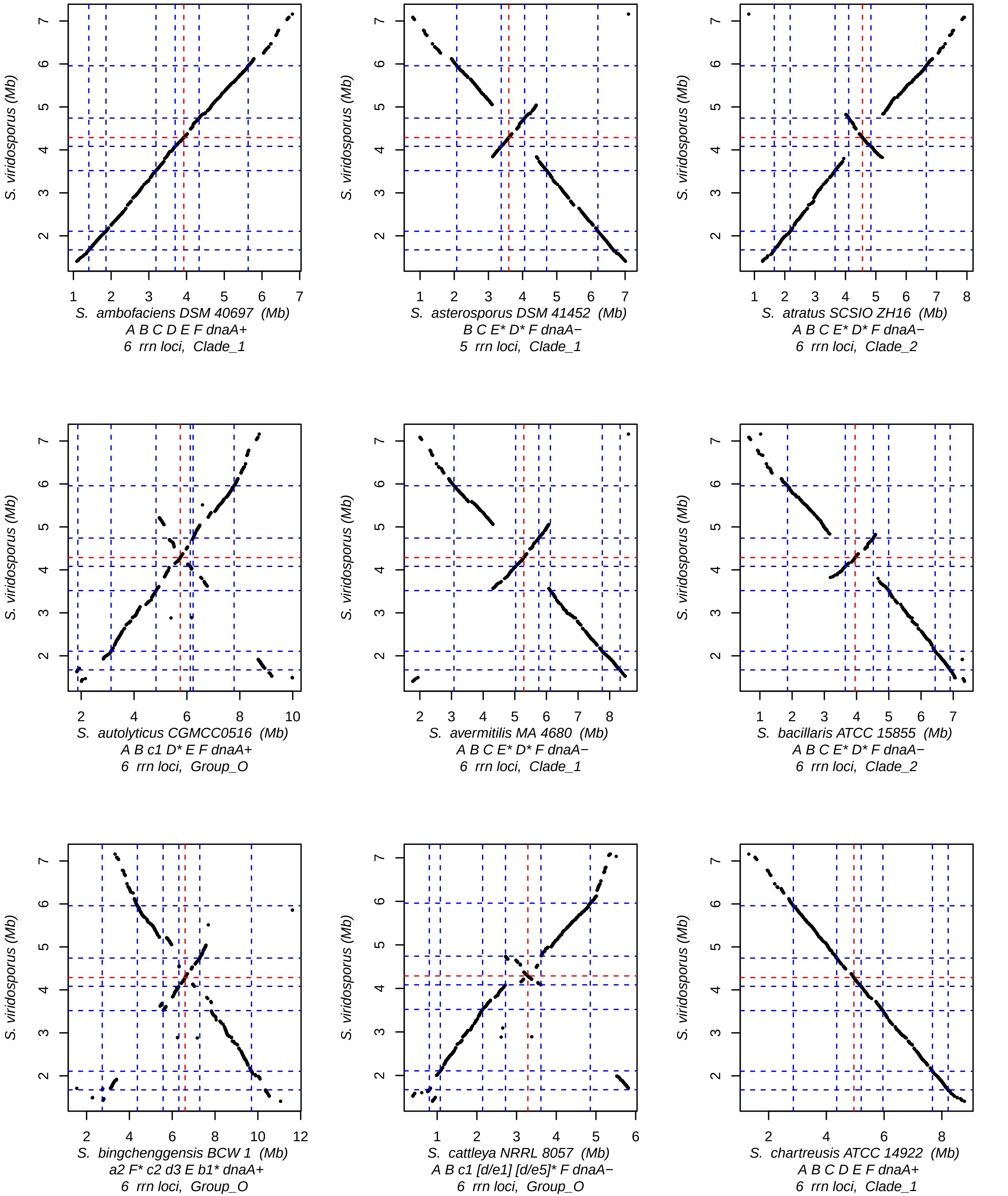

Figure S2 (3/15)

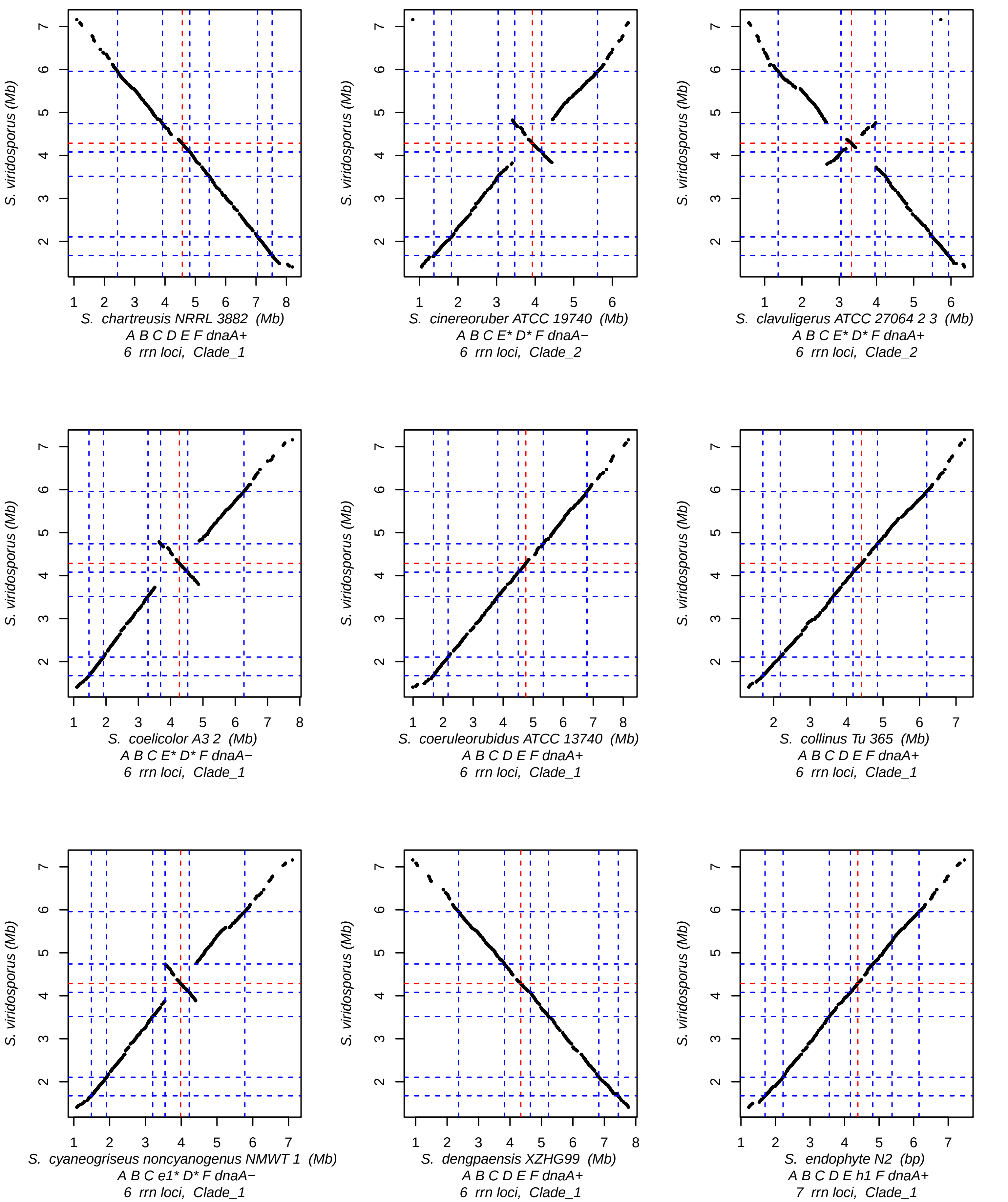

Figure S2 (4/15)

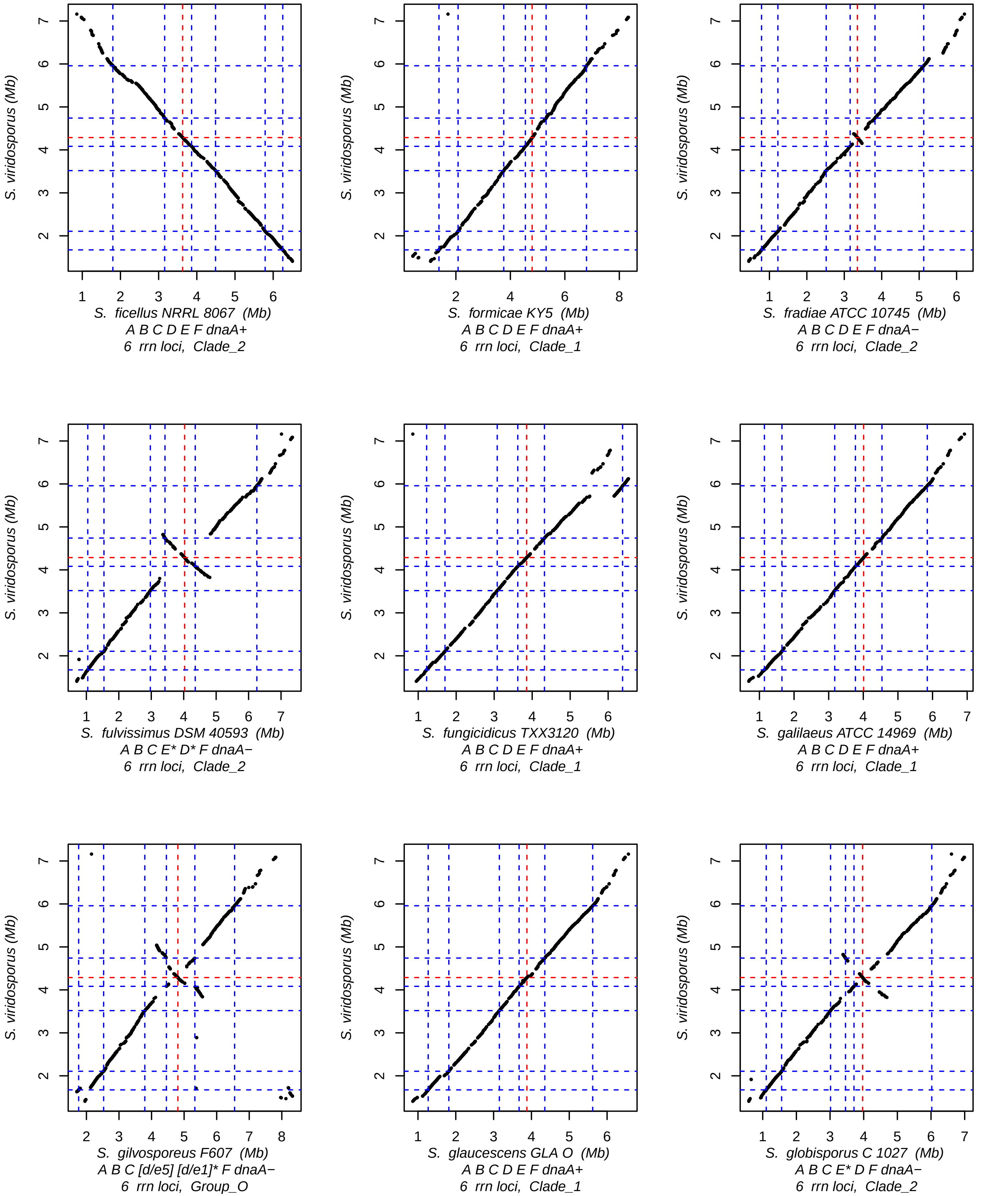

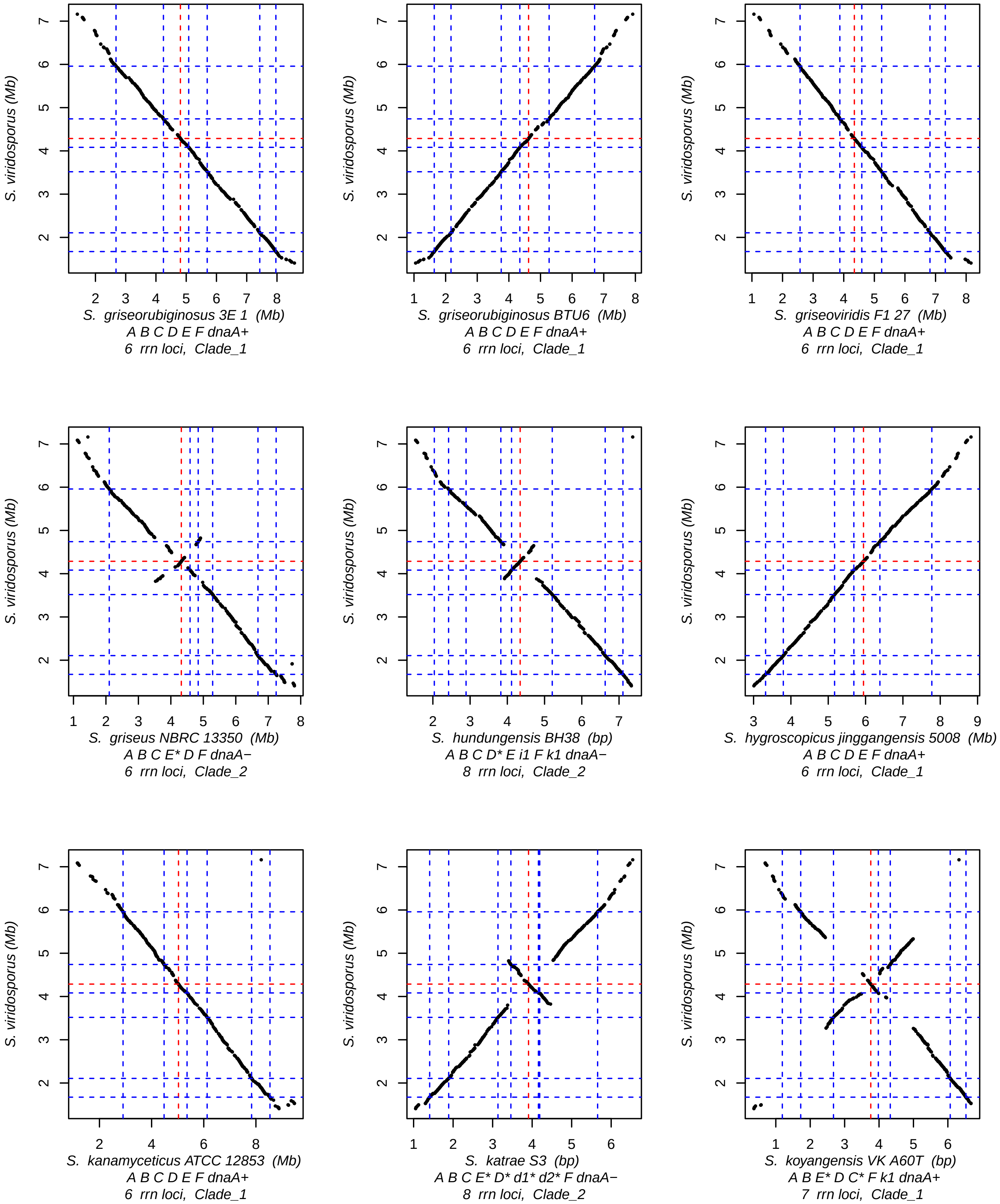
S. viridosporus (Mb)
S. griseorubiginosus 3E 1 (Mb)
A B C D E F dnaA+
6 rrn loci, Clade_1
S. griseorubiginosus BTU6 (Mb)
A B C D E F dnaA+
6 rrn loci, Clade_1
S. griseoviridis F1 27 (Mb)
A B C D E F dnaA+
6 rrn loci, Clade_1
S. griseus NBRC 13350 (Mb)
A B C E* D F dnaA−
6 rrn loci, Clade_2
S. hundungensis BH38 (bp)
A B C D* E i1 F k1 dnaA−
8 rrn loci, Clade_2
S. hygroscopicus jinggangensis 5008 (Mb)
A B C D E F dnaA+
6 rrn loci, Clade_1
S. kanamyceticus ATCC 12853 (Mb)
A B C D E F dnaA+
6 rrn loci, Clade_1
S. katrae S3 (bp)
A B C E* D* d1* d2* F dnaA−
8 rrn loci, Clade_2
S. koyangensis VK A60T (bp)
A B E* D C* F k1 dnaA+
7 rrn loci, Clade_1

Figure S2 (6/15)

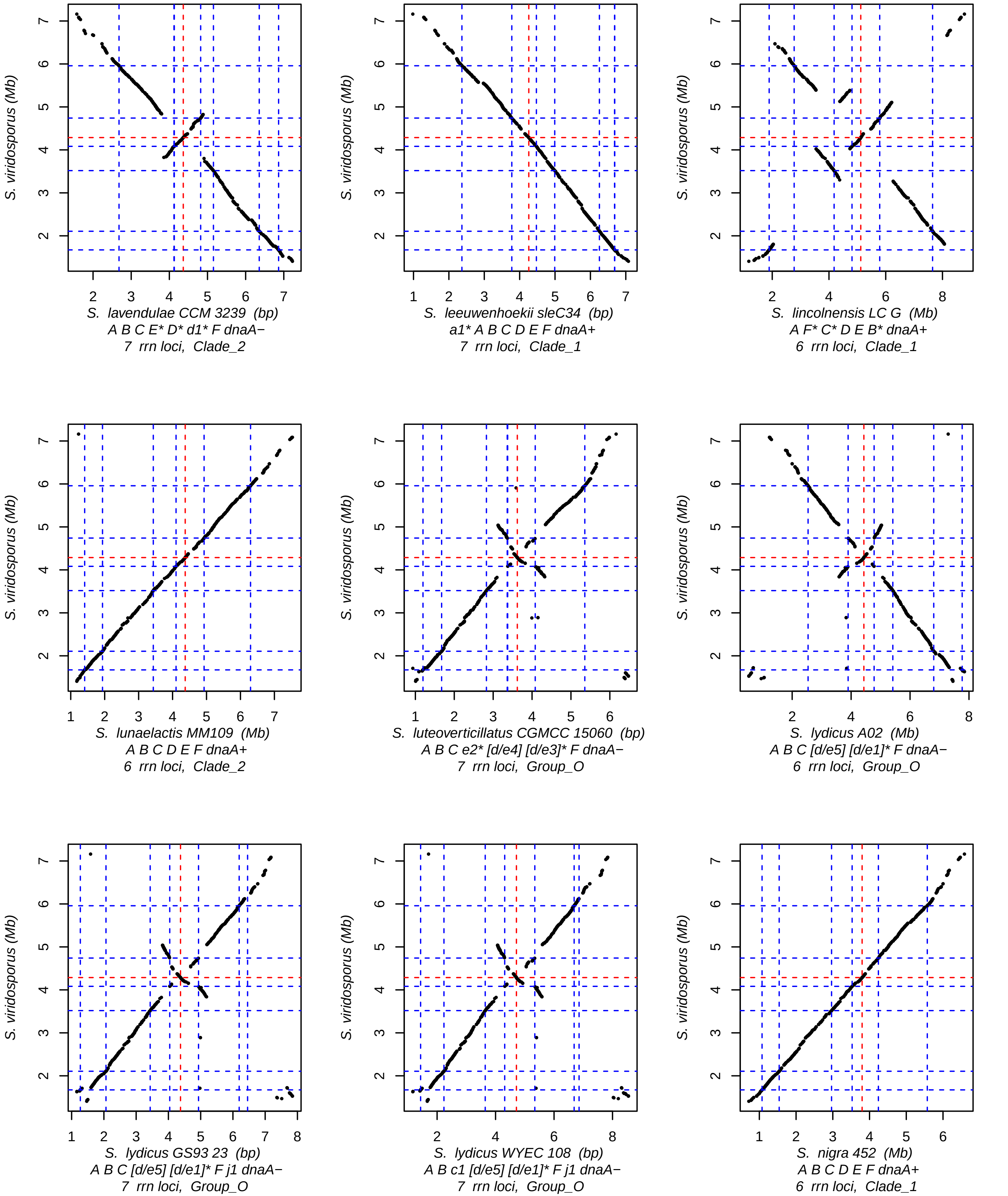

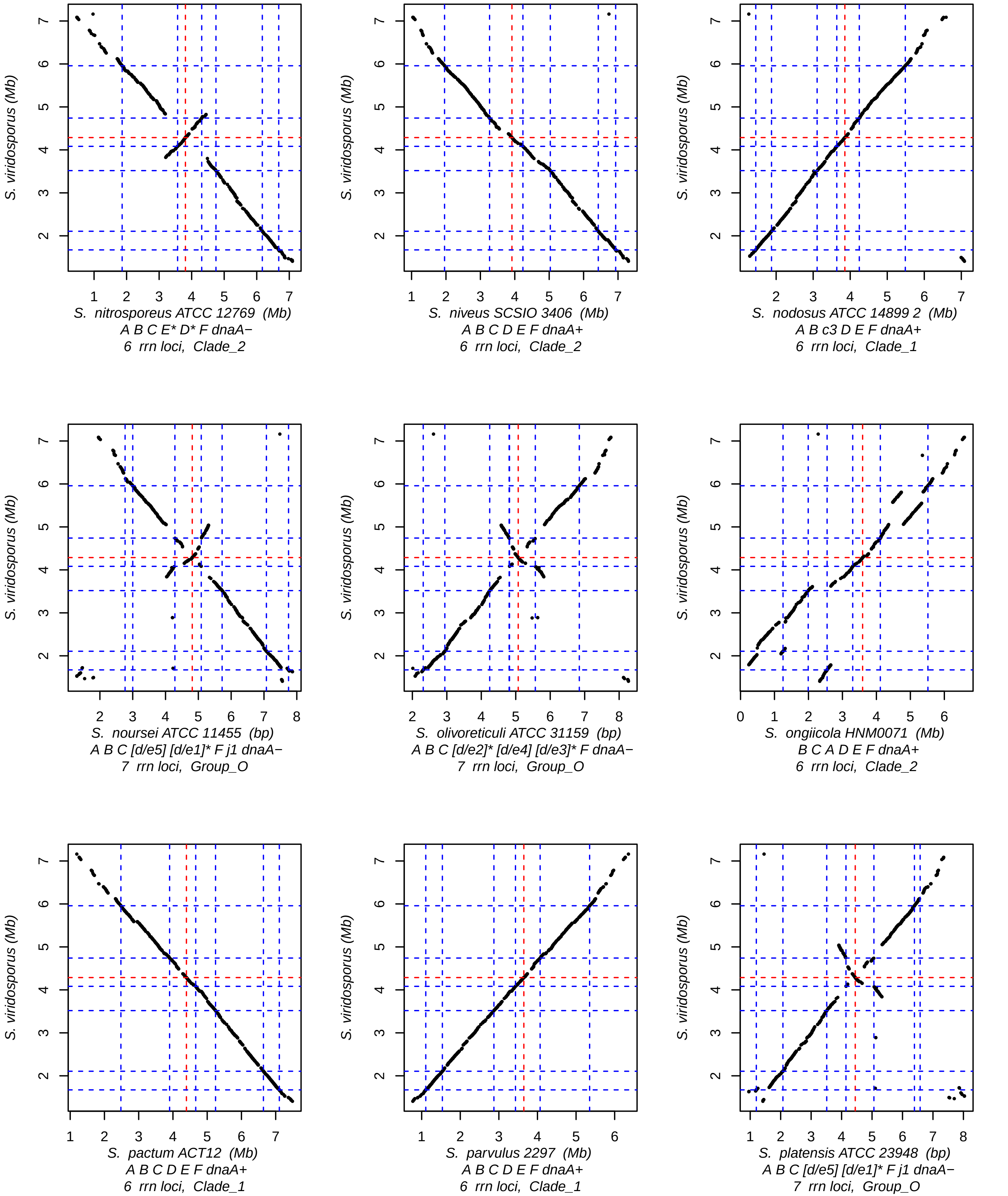
S. viridosporus (Mb)
S. nitrosporeus ATCC 12769 (Mb)
A B C E* D* F dnaA−
6 rrn loci, Clade_2
S. niveus SCSIO 3406 (Mb)
A B C D E F dnaA+
6 rrn loci, Clade_2
S. nodosus ATCC 14899 2 (Mb)
A B c3 D E F dnaA+
6 rrn loci, Clade_1
S. noursei ATCC 11455 (bp)
A B C [d/e5] [d/e1]* F j1 dnaA−
7 rrn loci, Group_O
S. olivoreticuli ATCC 31159 (bp)
A B C [d/e2]* [d/e4] [d/e3]* F dnaA−
7 rrn loci, Group_O
S. ongiicola HNM0071 (Mb)
B C A D E F dnaA+
6 rrn loci, Clade_2
S. pactum ACT12 (Mb)
A B C D E F dnaA+
6 rrn loci, Clade_1
S. parvulus 2297 (Mb)
A B C D E F dnaA+
6 rrn loci, Clade_1
S. platensis ATCC 23948 (bp)
A B C [d/e5] [d/e1]* F j1 dnaA−
7 rrn loci, Group_O

Figure S2 (8/15)

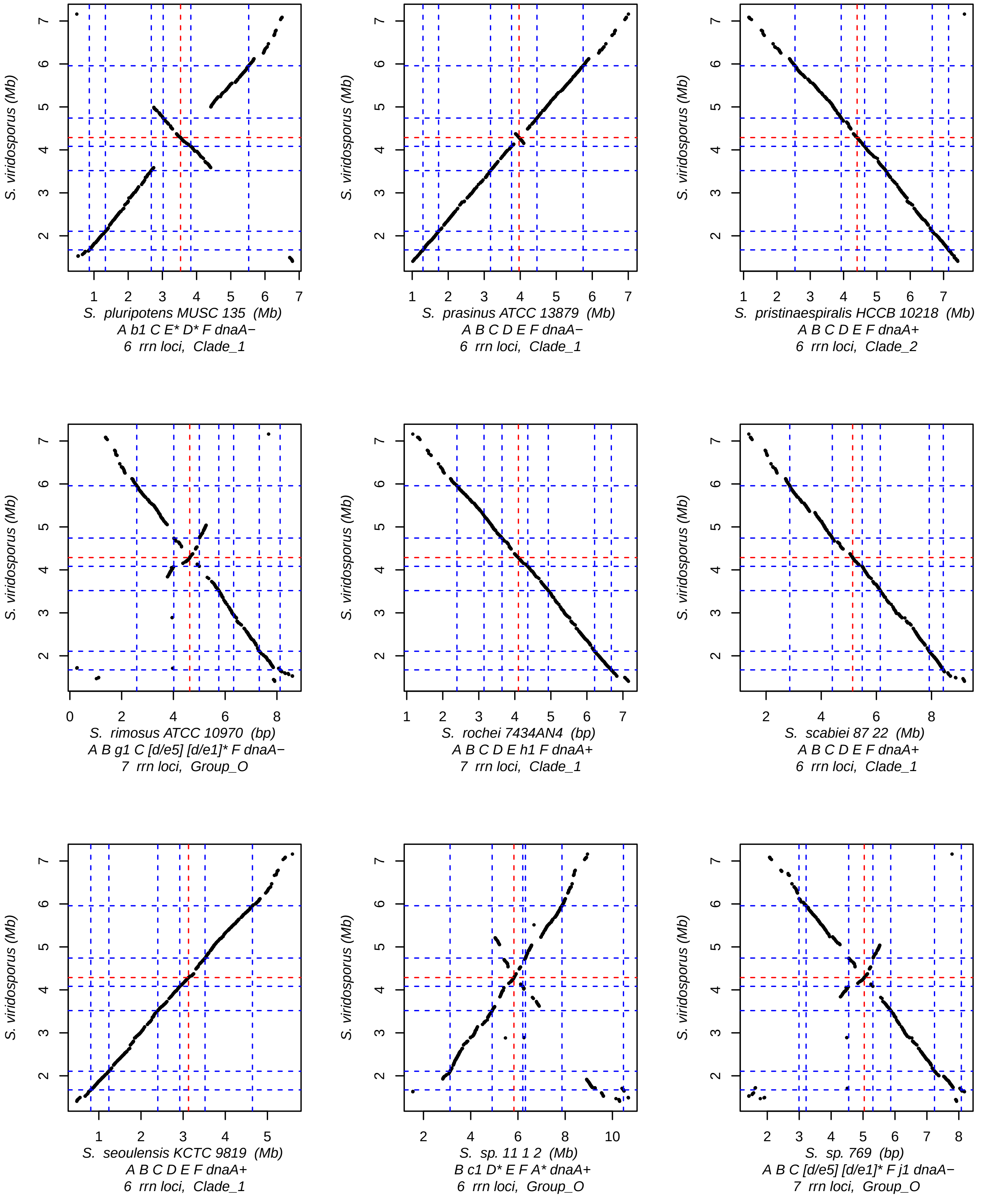

Figure S2 (9/15)

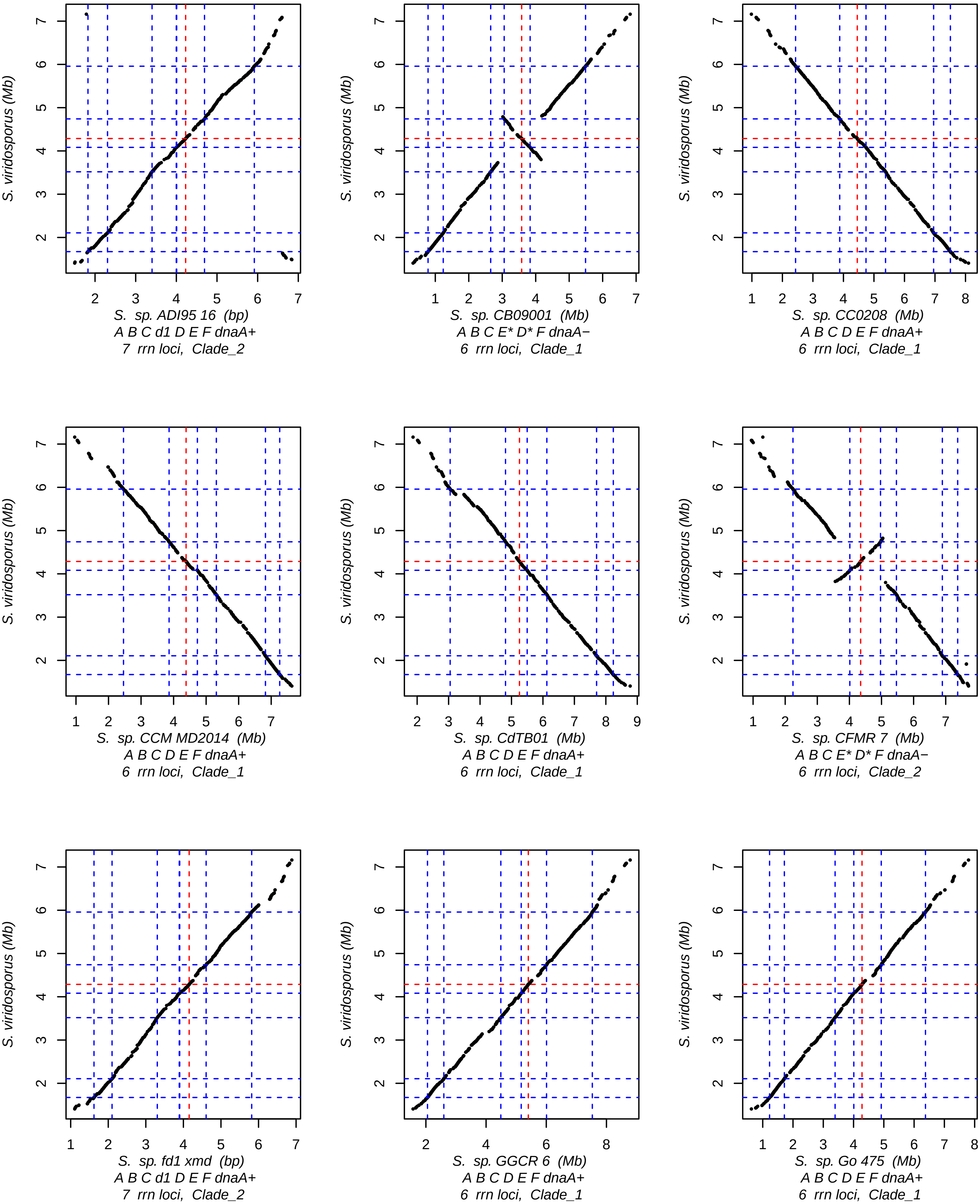

Figure S2 (10/15)

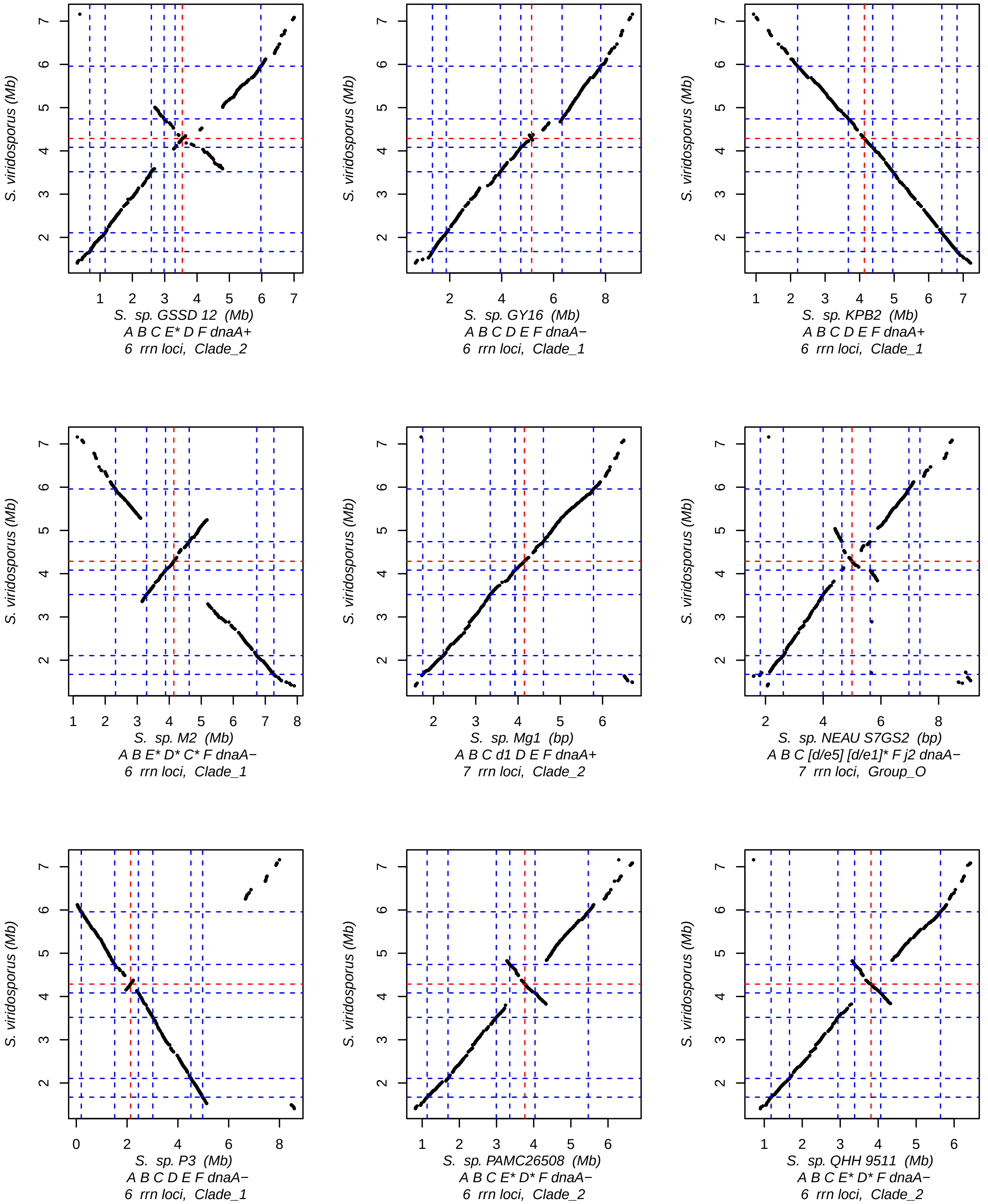

Figure S2 (11/15)

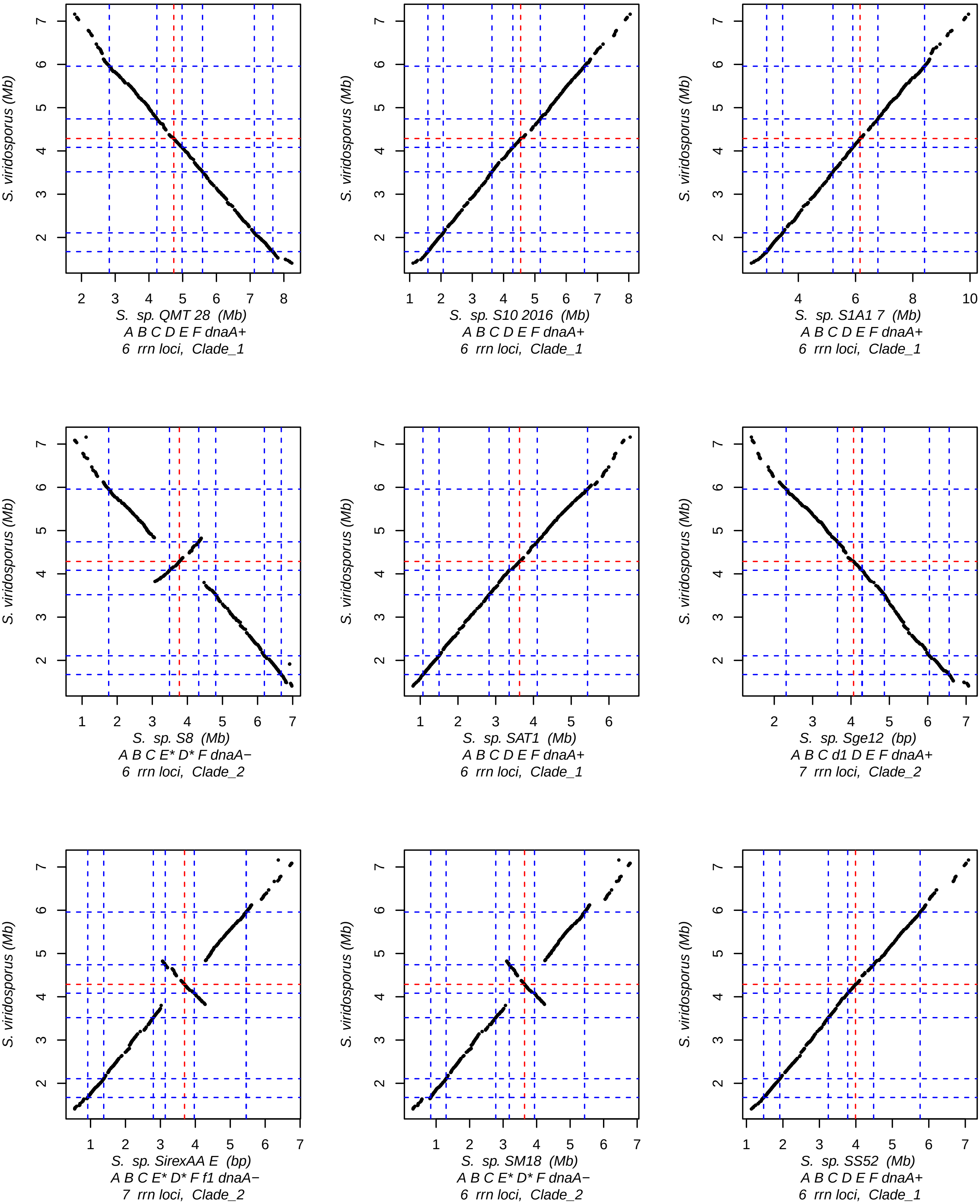

Figure S2 (12/15)

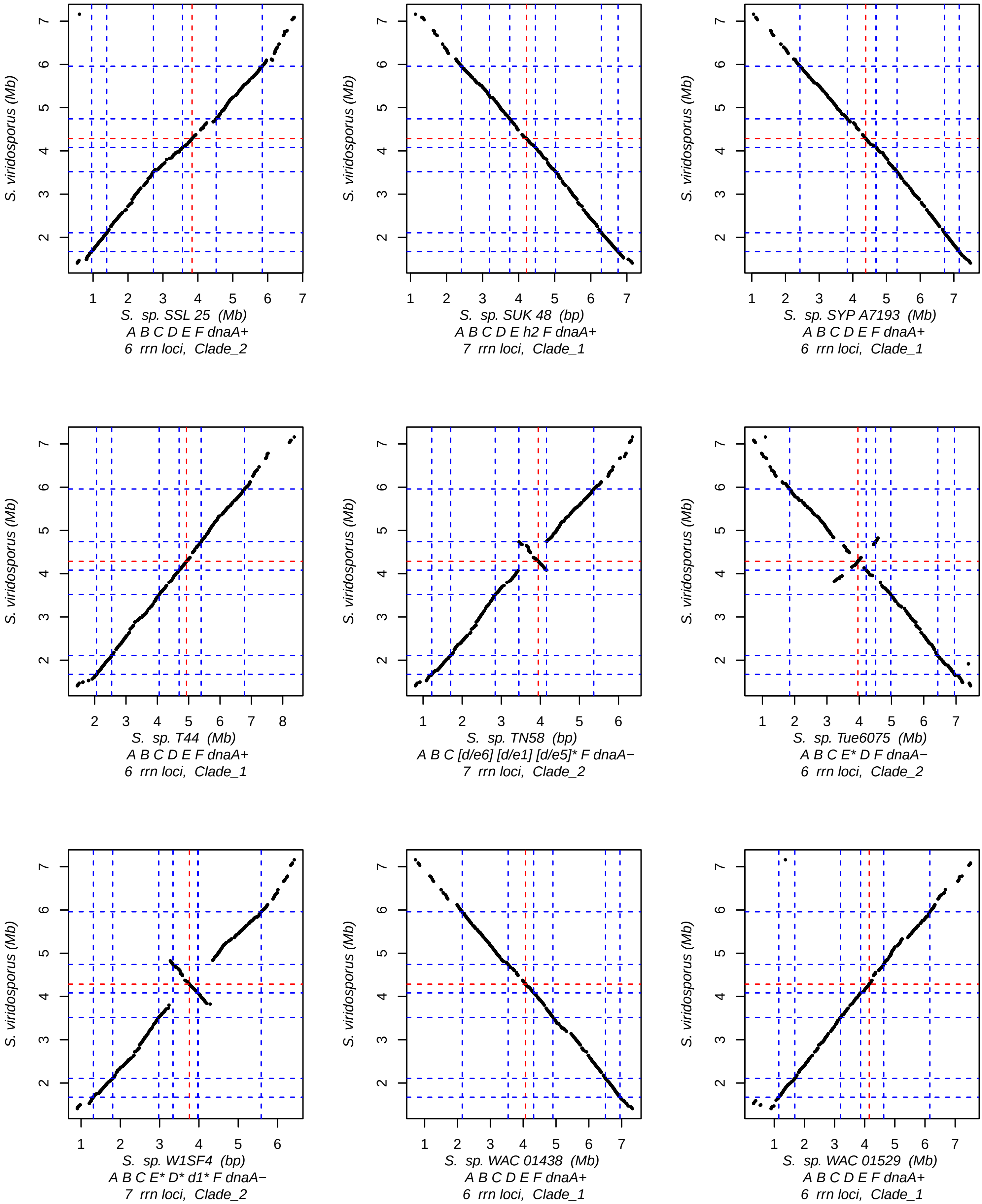

Figure S2 (13/15)

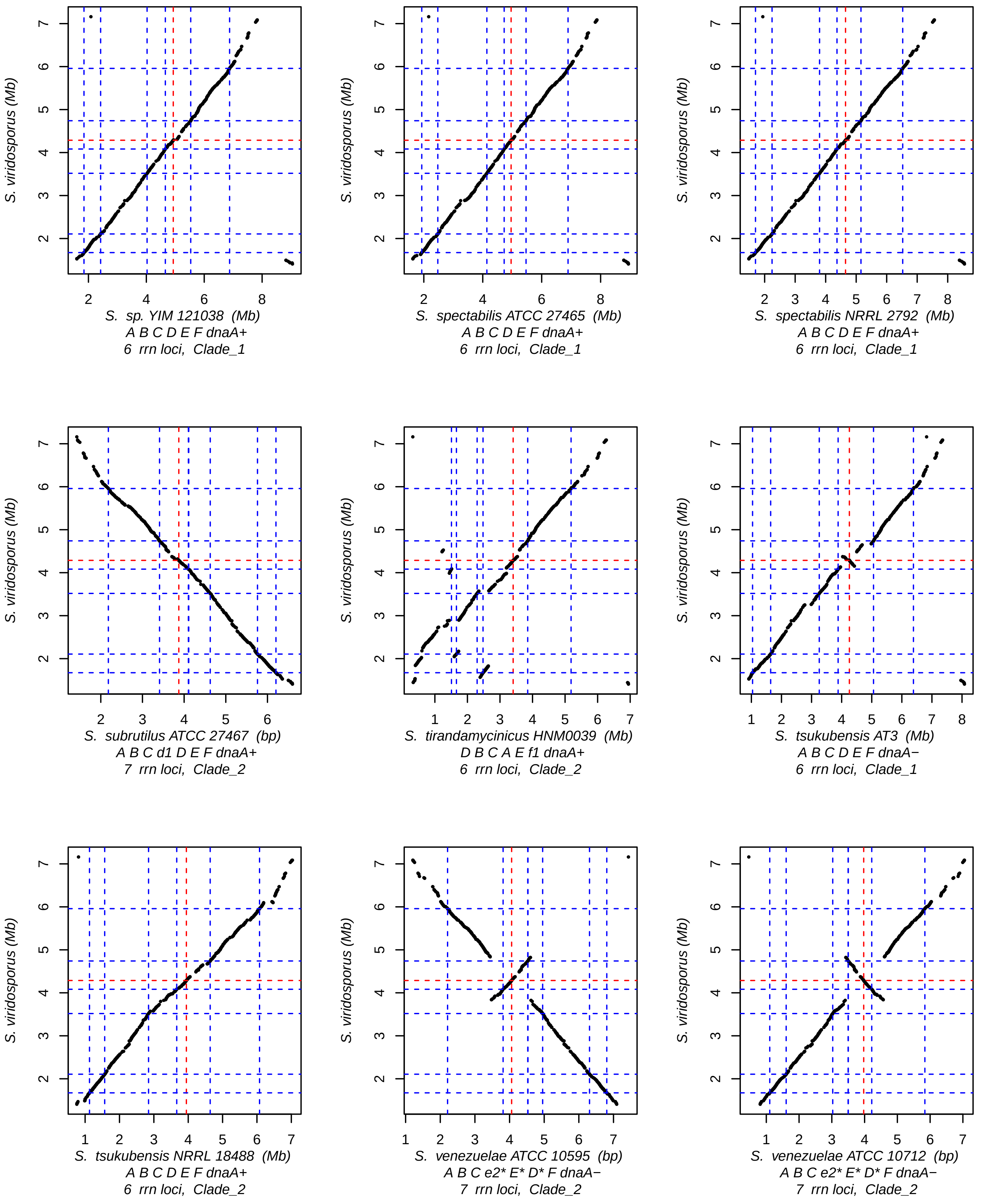

Figure S2 (14/15)

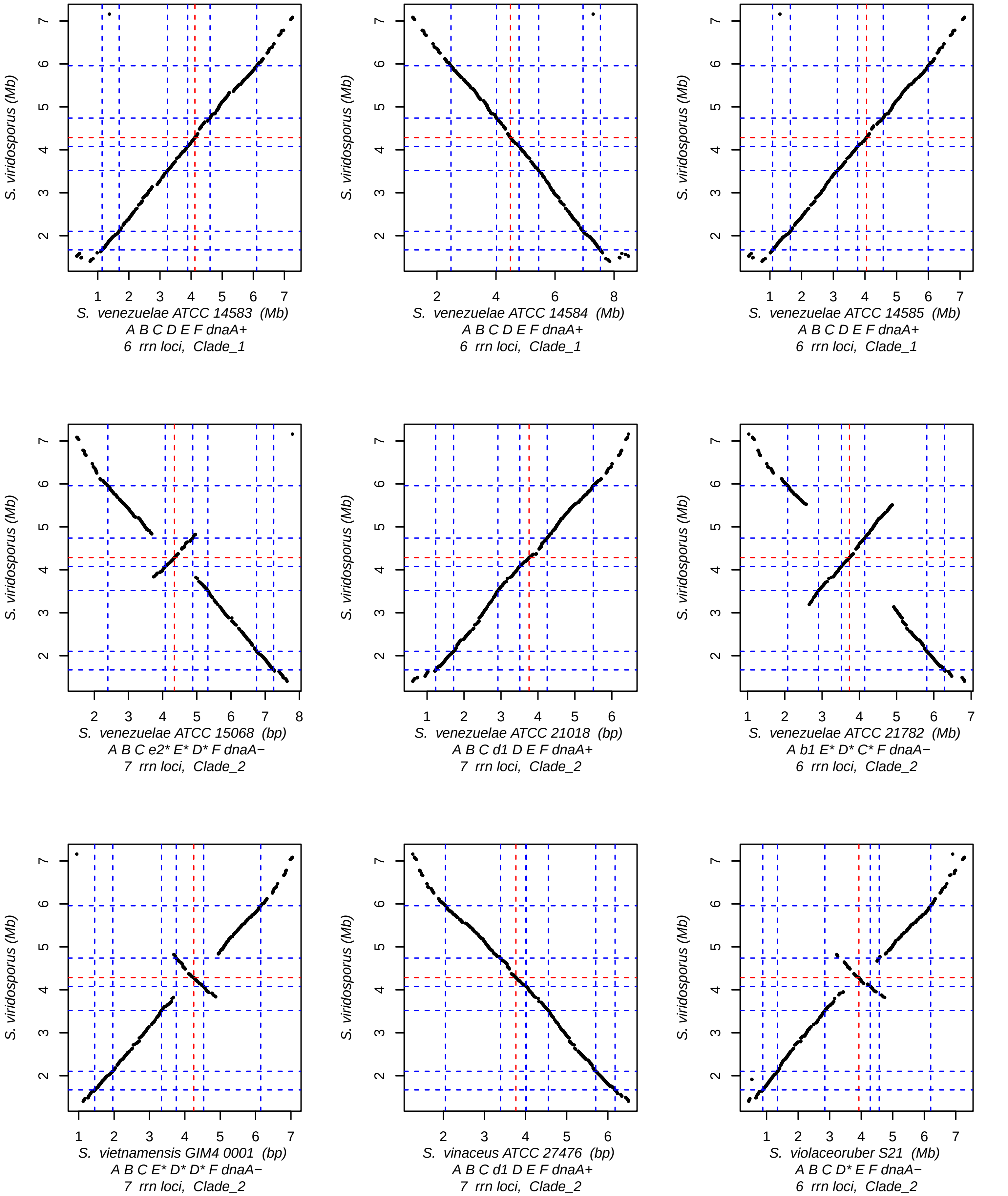

Figure S2: Pairwise comparisons of all the core genomes of the panel with the consensus reference strain, *Streptomyces viridosporus* T7A ATCC 39115

The *rrn* nomenclature, number of *rrn*, and clade (or group) are indicated for each strain below each graph. The positions of *rrn* operons and of the origin of replication are indicated by blue and red dashed lines, respectively. The presence of an unbroken diagonal indicates that the strains have identically ordered core genomes. When the diagonal starts at the bottom left and ends at the top right, it means that the genome sequence available in the databases is oriented in the same orientation as the consensus used in this study (*Streptomyces viridosporus* T7A ATCC 39115), and that the *rrn* configuration shown below each graph can be directly transposed onto the graph. If not, the sequence is in the opposite direction. In this case, the consensus should be reversed when transposed on the graph (e.g. '*rrn* ABCDEF *dnaA*+' becomes '*rrn* F*E*D*C*B*A* *dnaA*-').
