# Supplementary material for: Ribosomal RNA operons define a central functional compartment in the Streptomyces chromosome
Source: Nucleic Acids Res. 2022 Nov 21;50(20):11654–69. doi: 10.1093/nar/gkac1076 (PMC9723626; doi:10.1093/nar/gkac1076)
Supplement: gkac1076_Supplemental_Files [file gkac1076_supplemental_files.zip › Supplementary_Figure_S5.pdf]

Figure S5 (1/15)

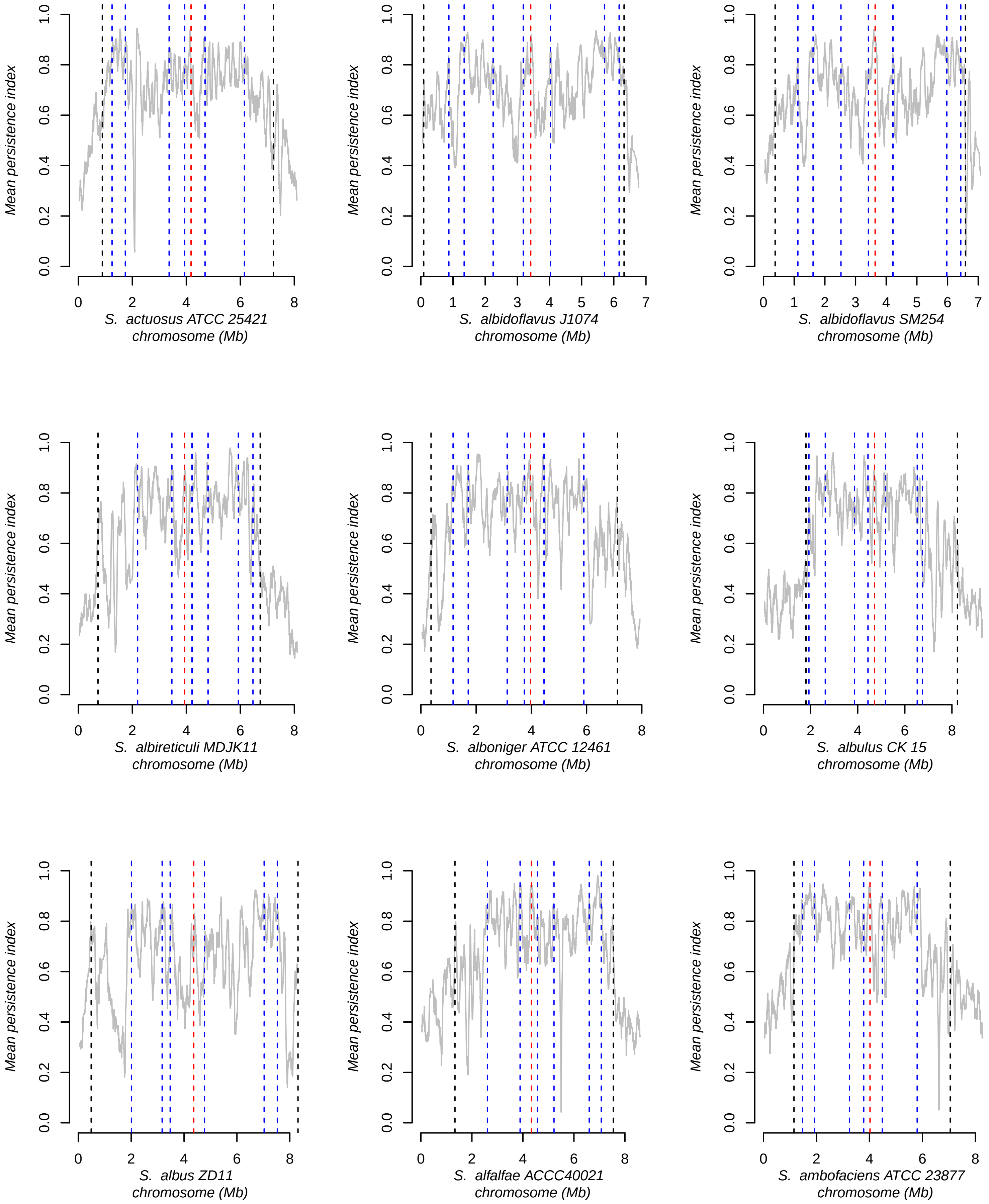

Figure S5 (2/15)

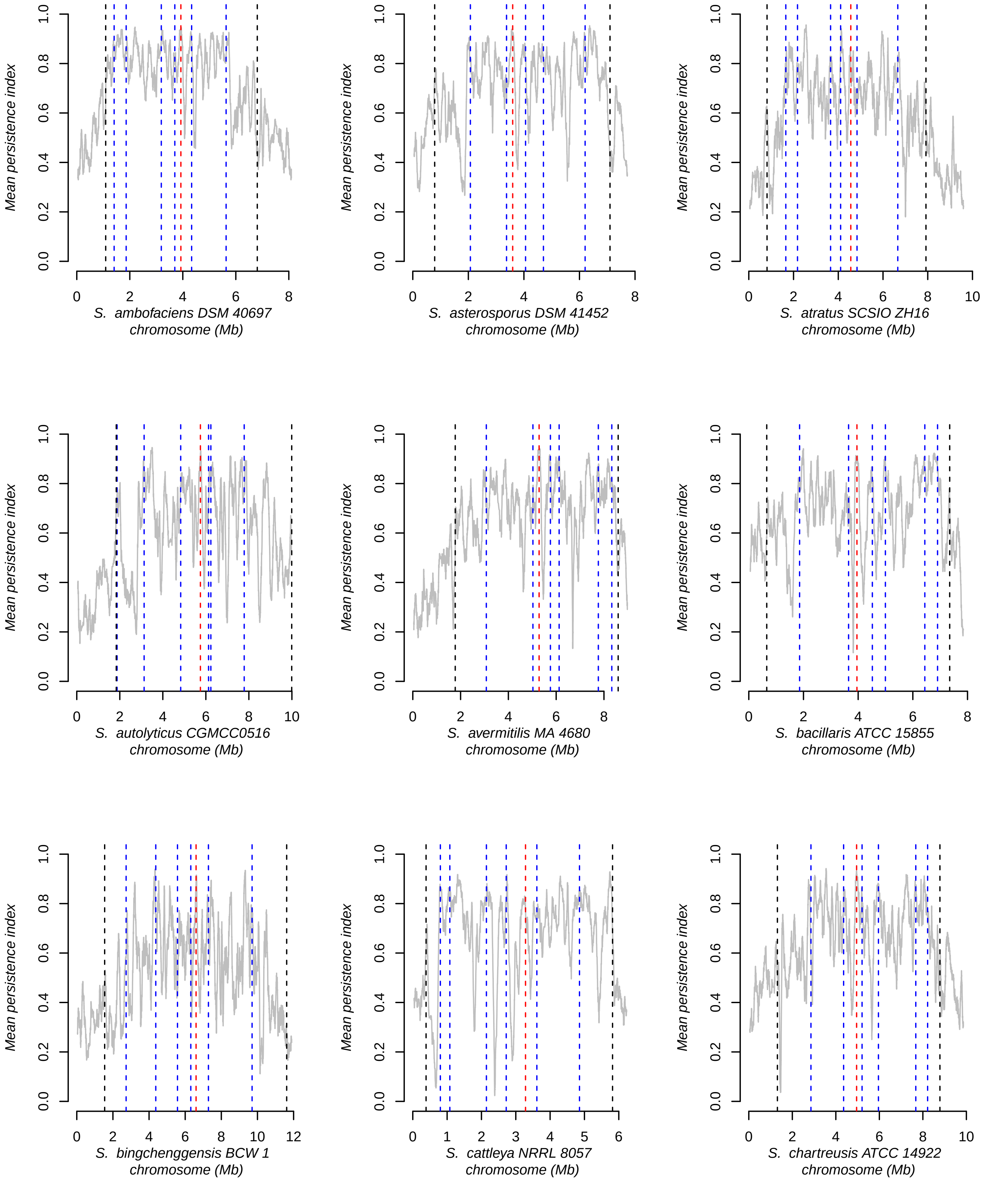

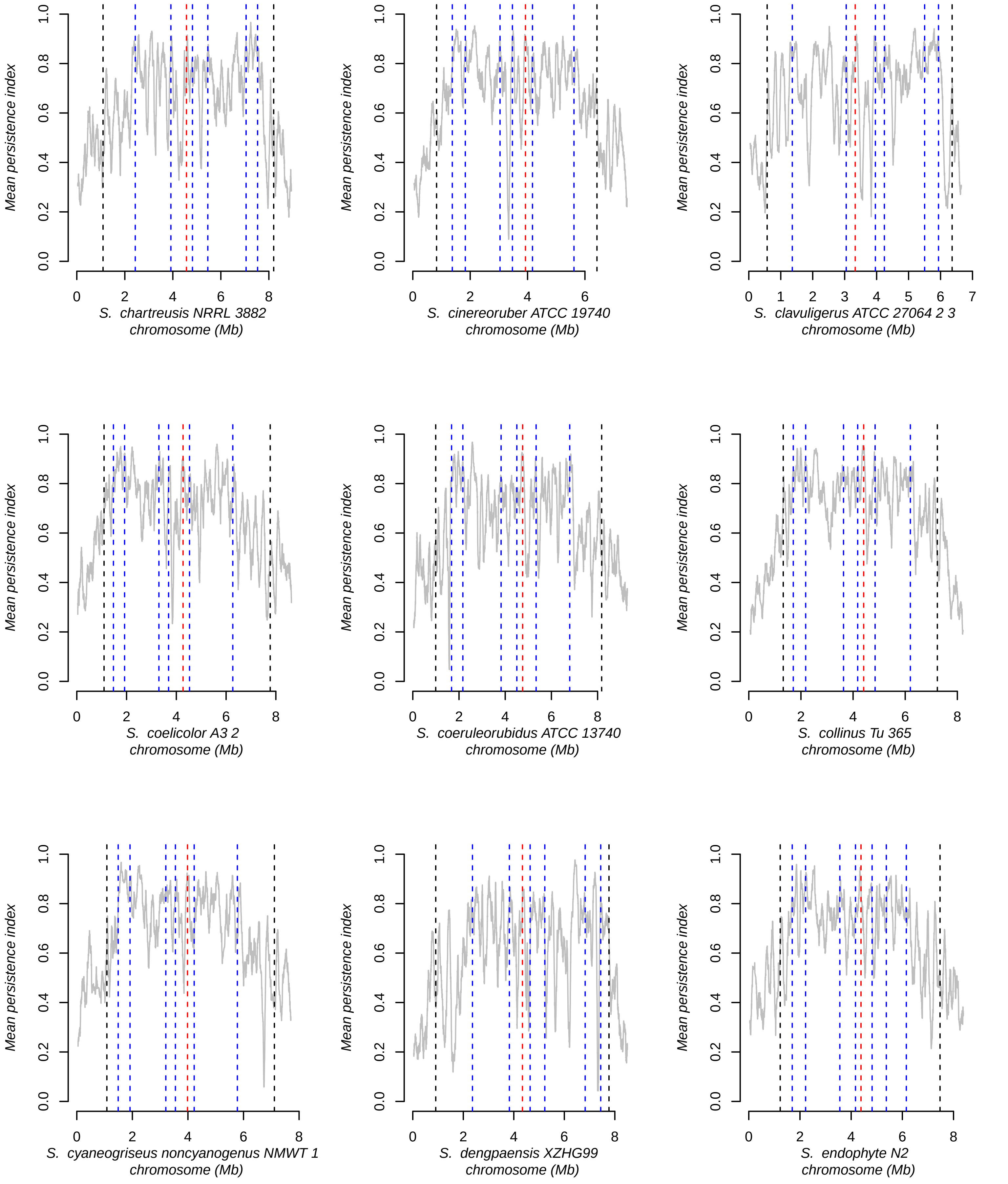
Mean persistence index
0.0
0.2
0.4
0.6
0.8
1.0
S. chartreusis NRRL 3882
chromosome (Mb)
S. cinereoruber ATCC 19740
chromosome (Mb)
S. clavuligerus ATCC 27064 2 3
chromosome (Mb)
S. coelicolor A3 2
chromosome (Mb)
S. coeruleorubidus ATCC 13740
chromosome (Mb)
S. collinus Tu 365
chromosome (Mb)
S. cyaneogriseus noncyanogenus NMWT 1
chromosome (Mb)
S. dengpaensis XZHG99
chromosome (Mb)
S. endophyte N2
chromosome (Mb)

Figure S5 (4/15)

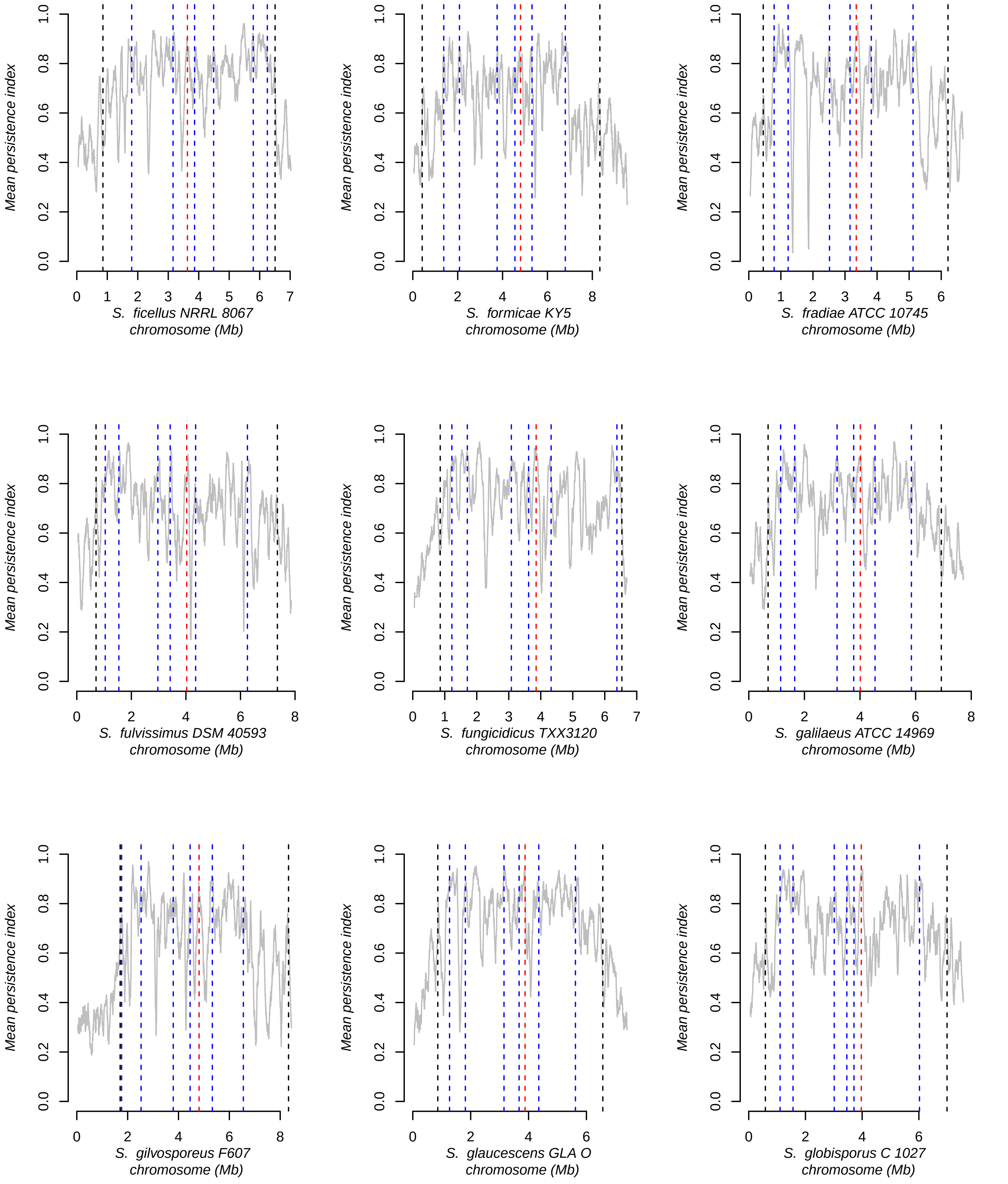

Figure S5 (5/15)

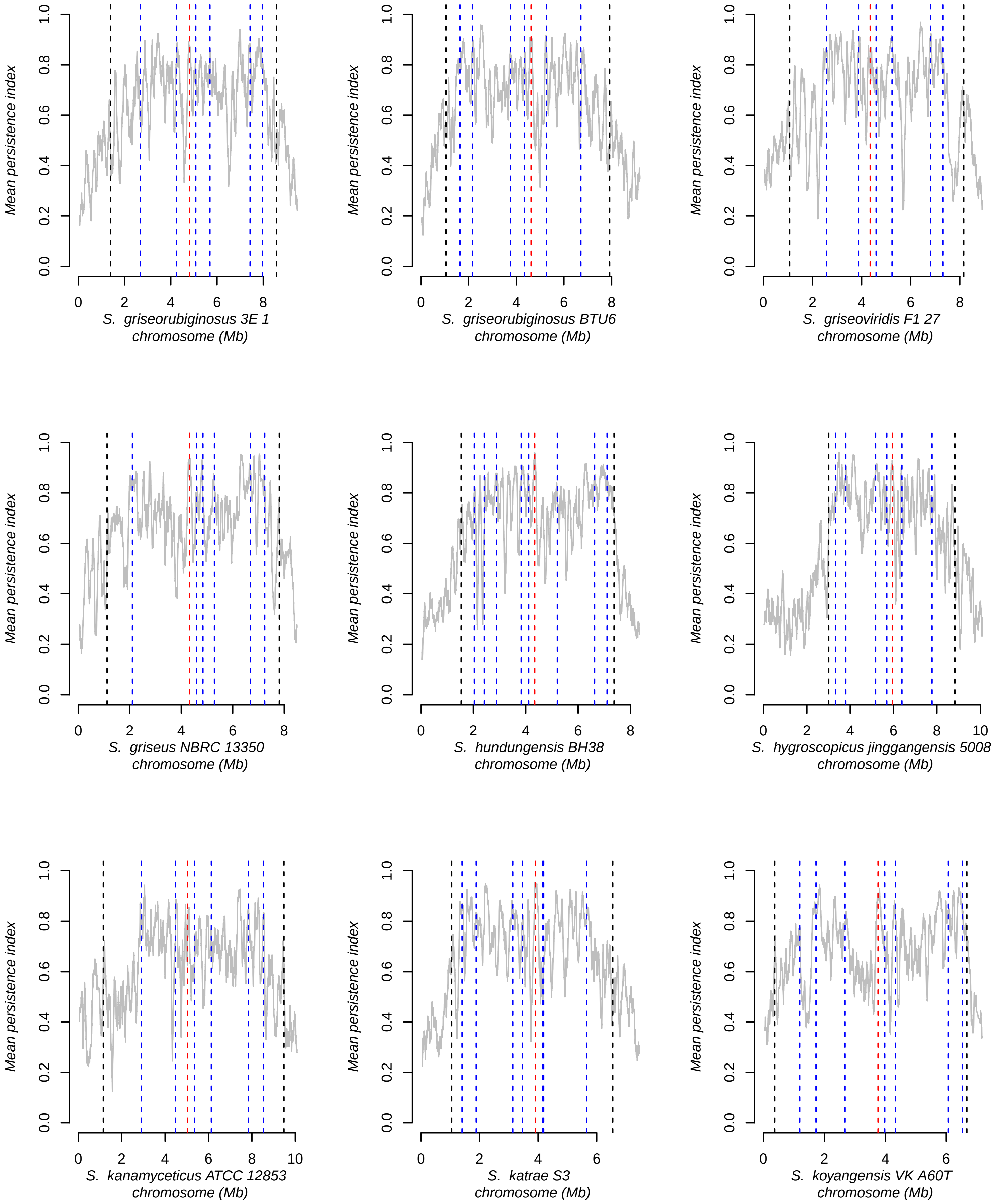

Figure S5 (6/15)

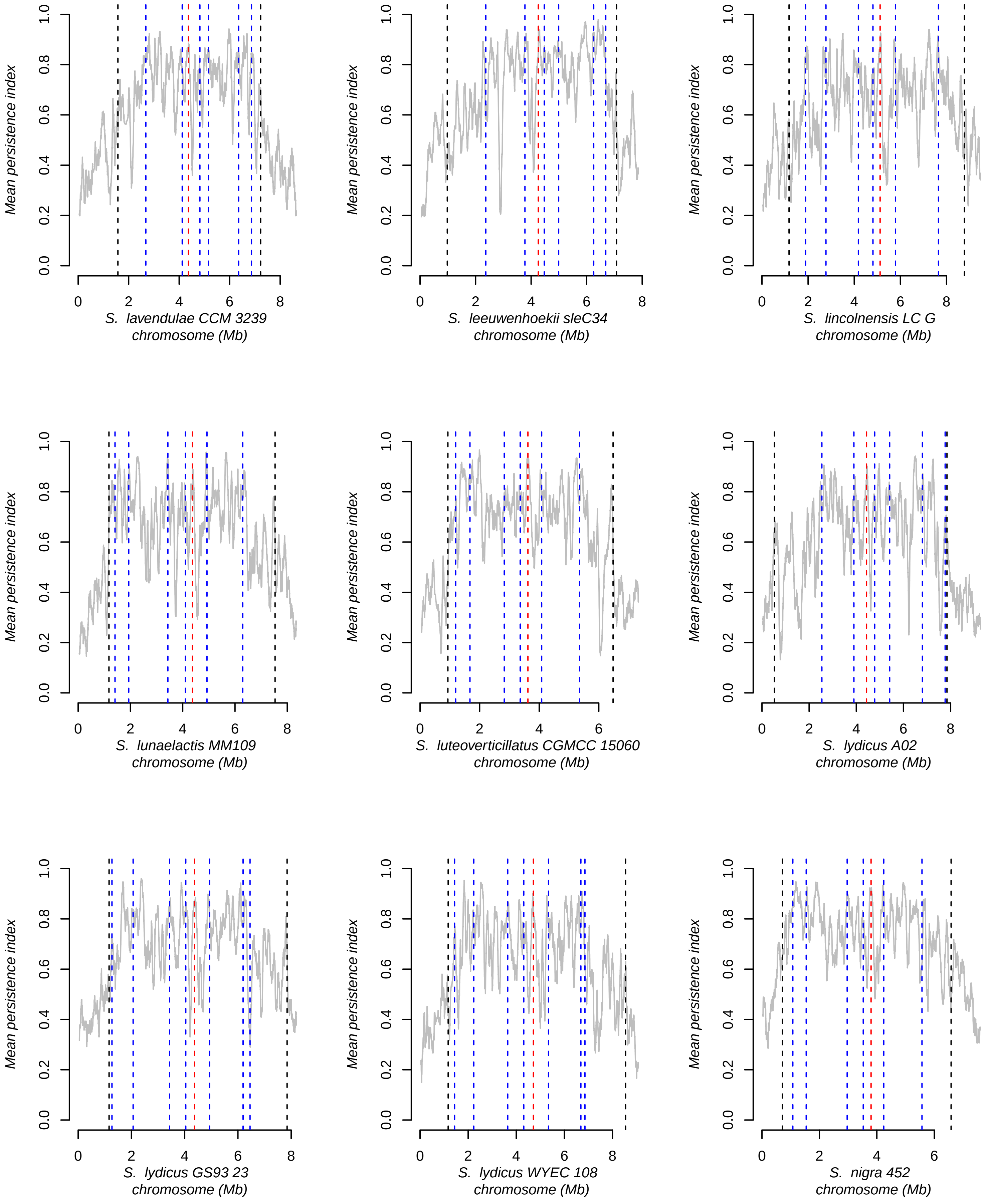

Figure S5 (7/15)

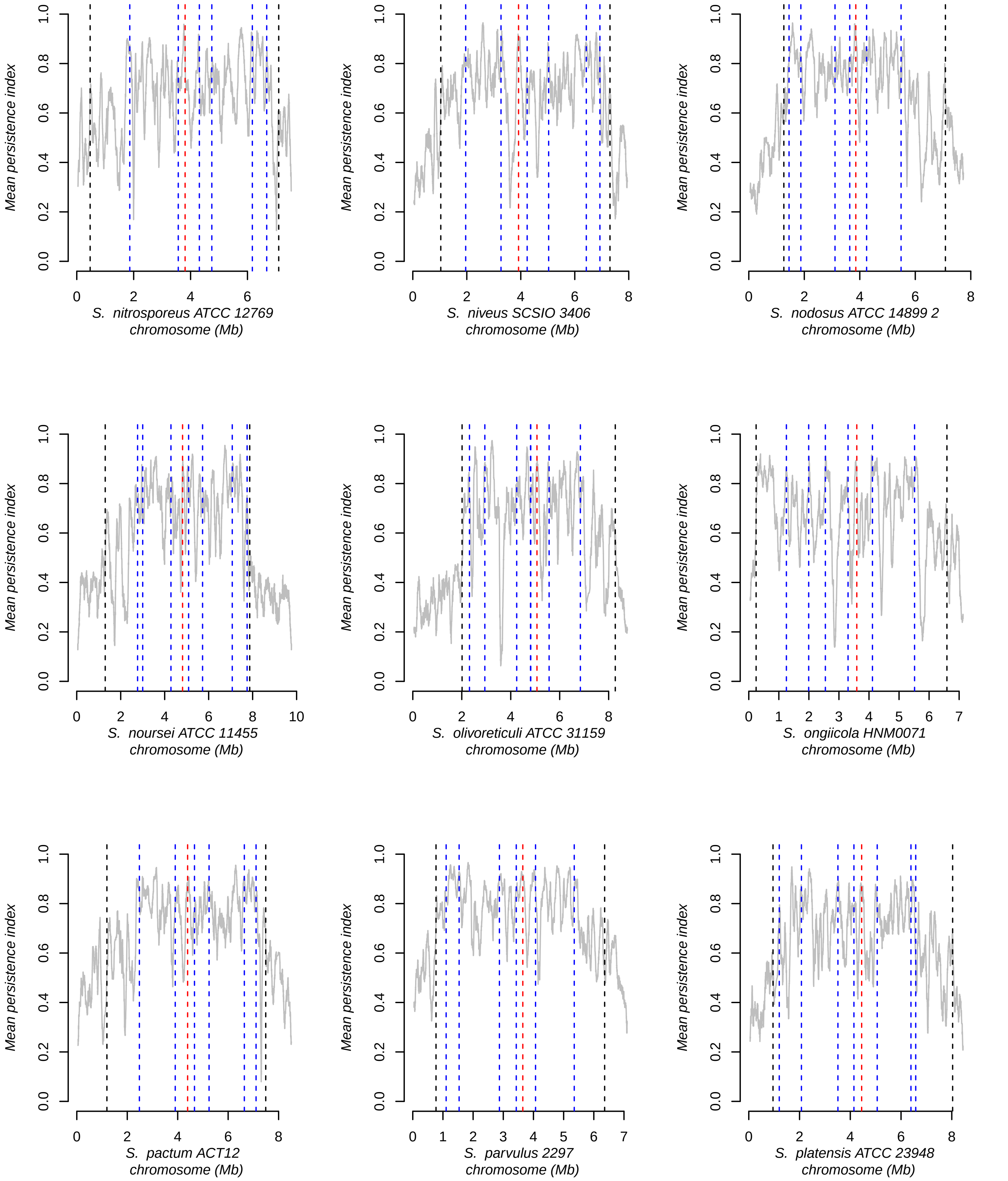

Figure S5 (8/15)

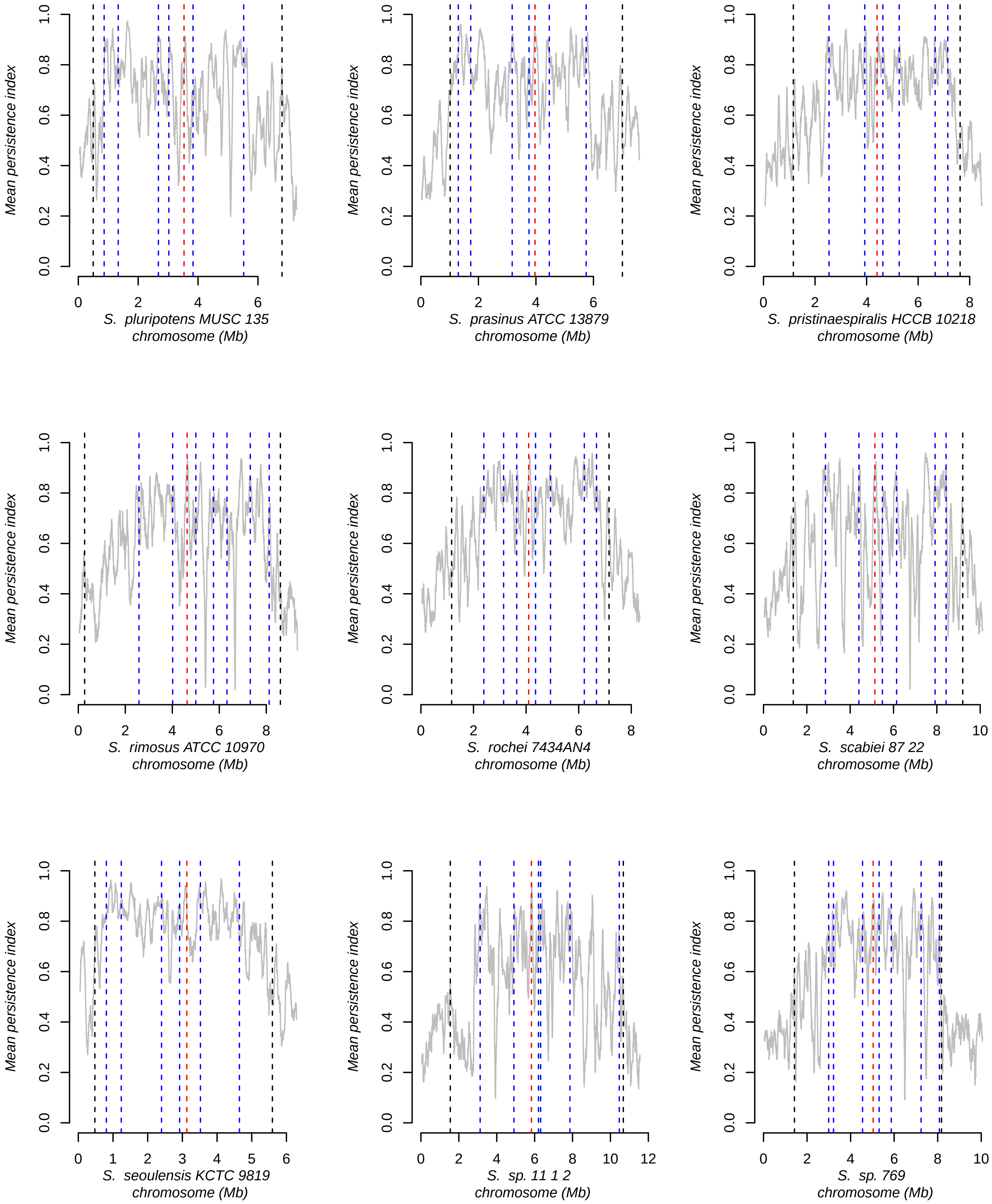

Figure S5 (9/15)

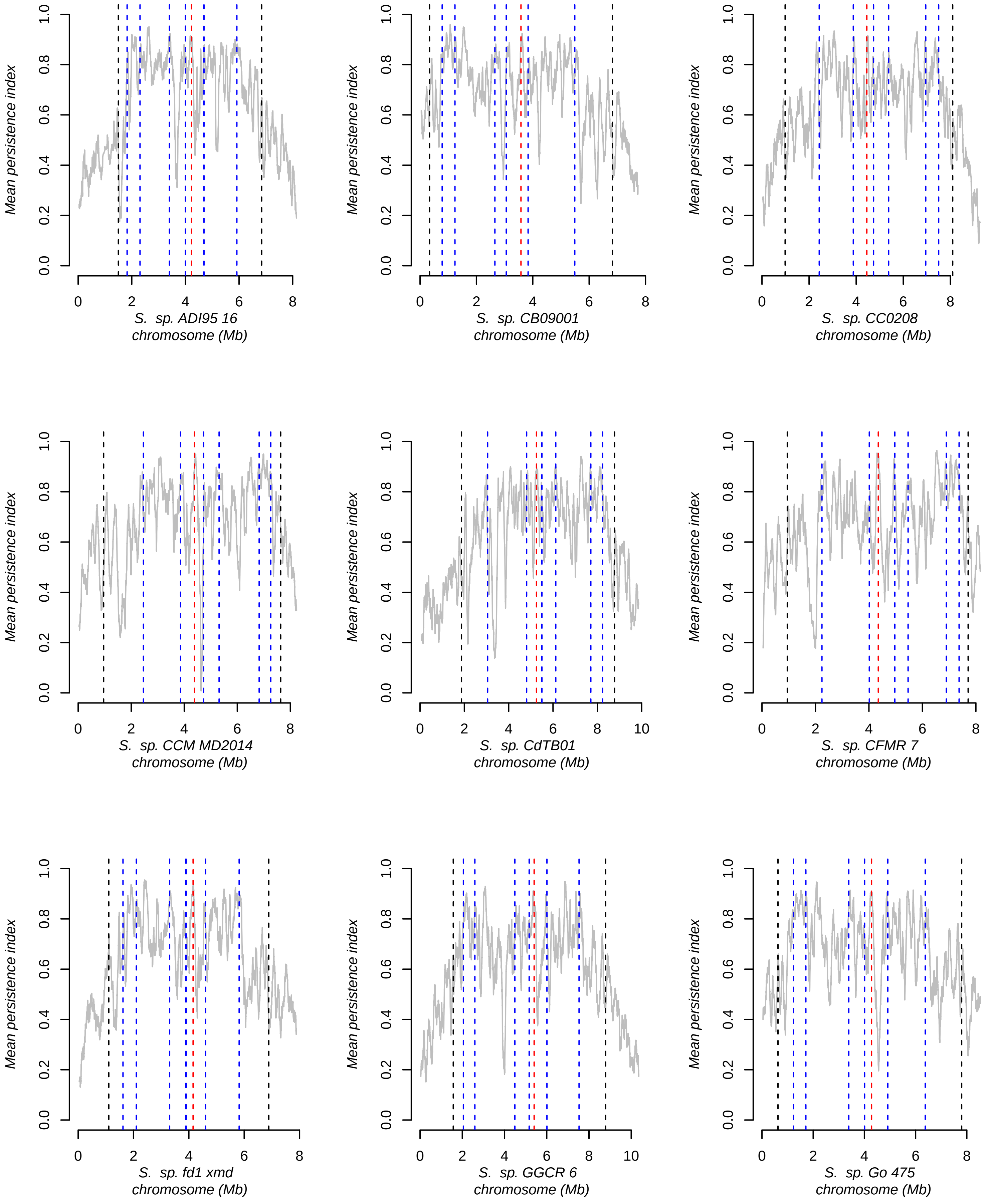

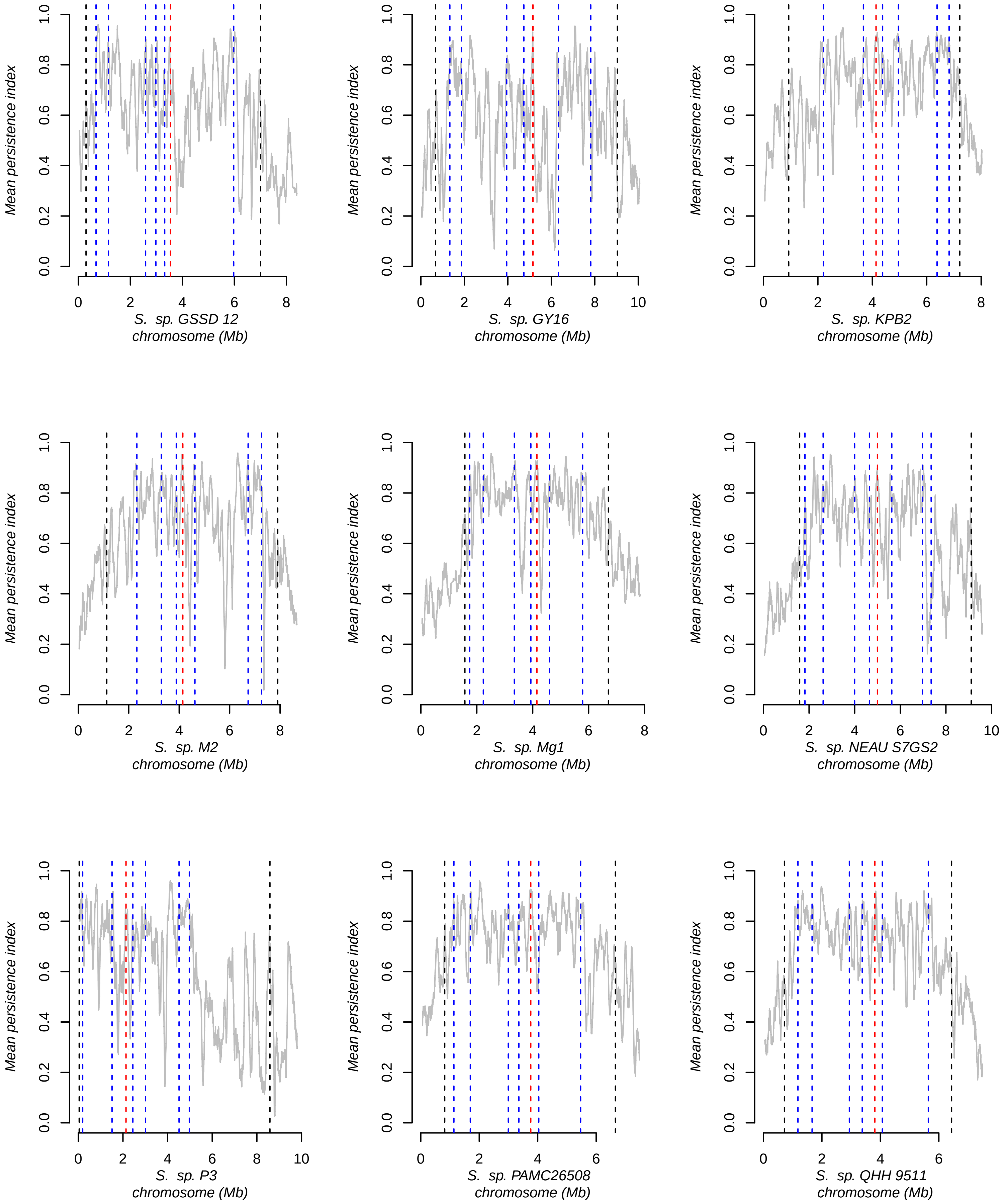
Mean persistence index
S. sp. GSSD 12 chromosome (Mb)
S. sp. GY16 chromosome (Mb)
S. sp. KPB2 chromosome (Mb)
S. sp. M2 chromosome (Mb)
S. sp. Mg1 chromosome (Mb)
S. sp. NEAU S7GS2 chromosome (Mb)
S. sp. P3 chromosome (Mb)
S. sp. PAMC26508 chromosome (Mb)
S. sp. QHH 9511 chromosome (Mb)

Figure S5 (11/15)

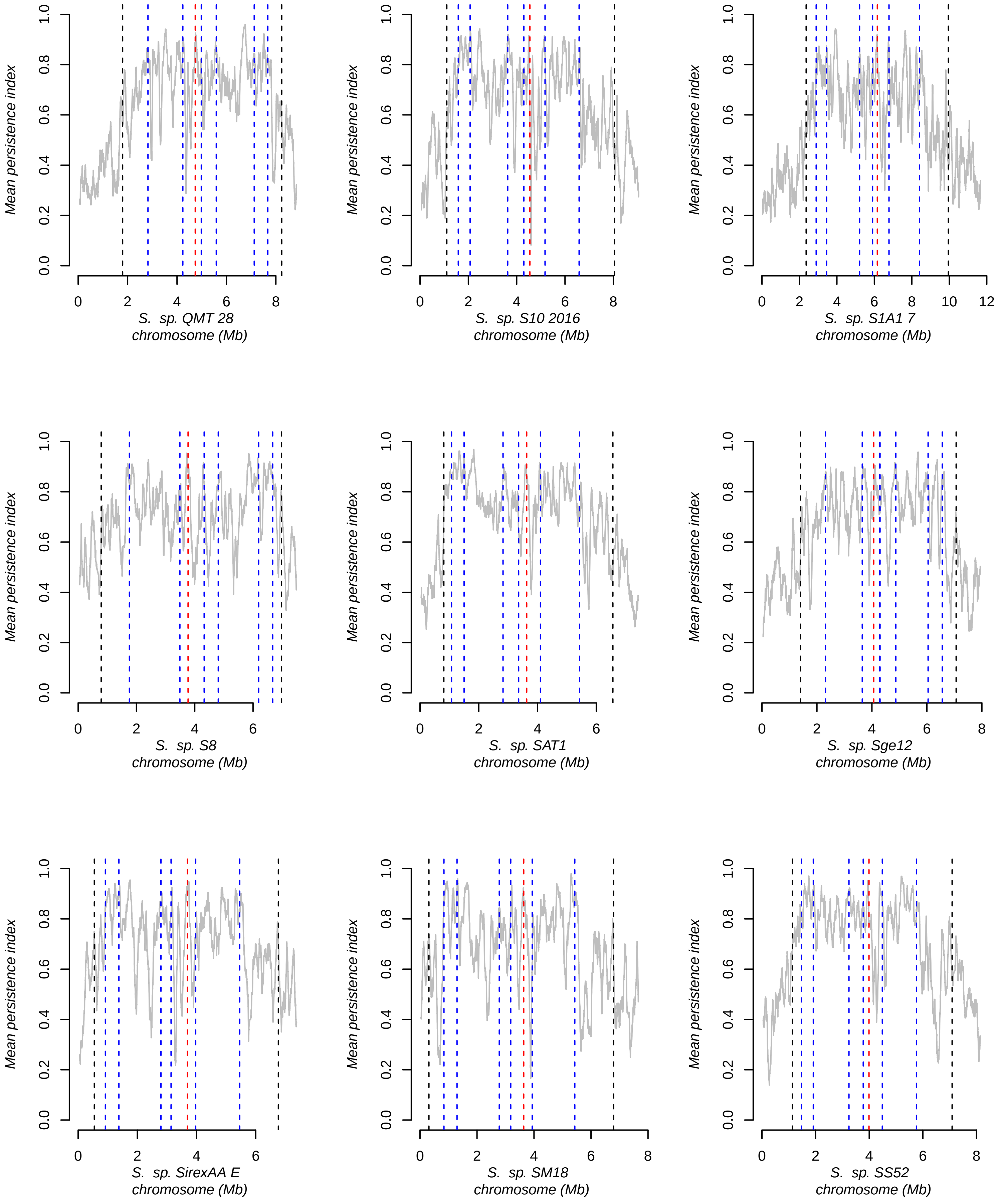

Figure S5 (12/15)

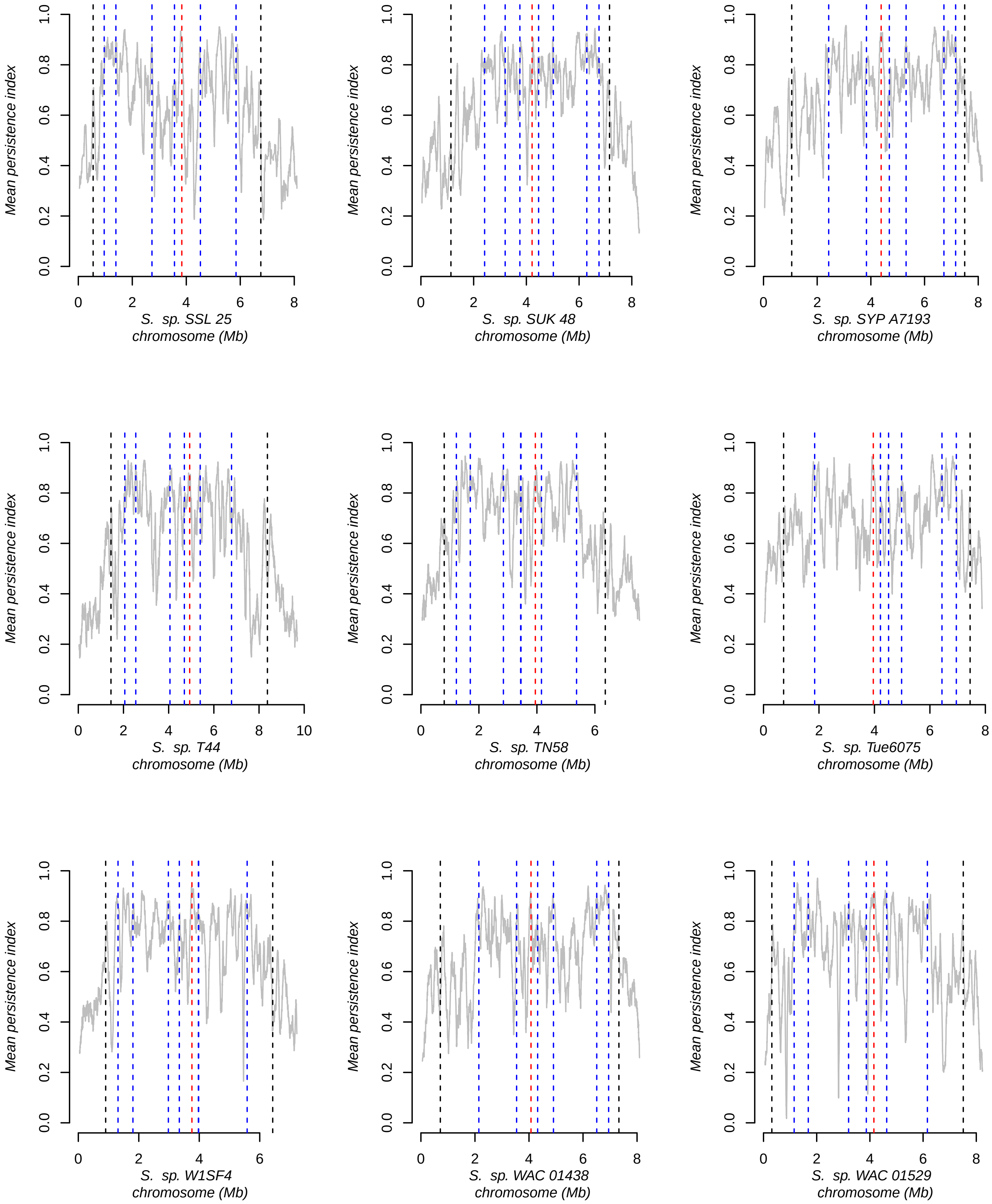

Figure S5 (13/15)

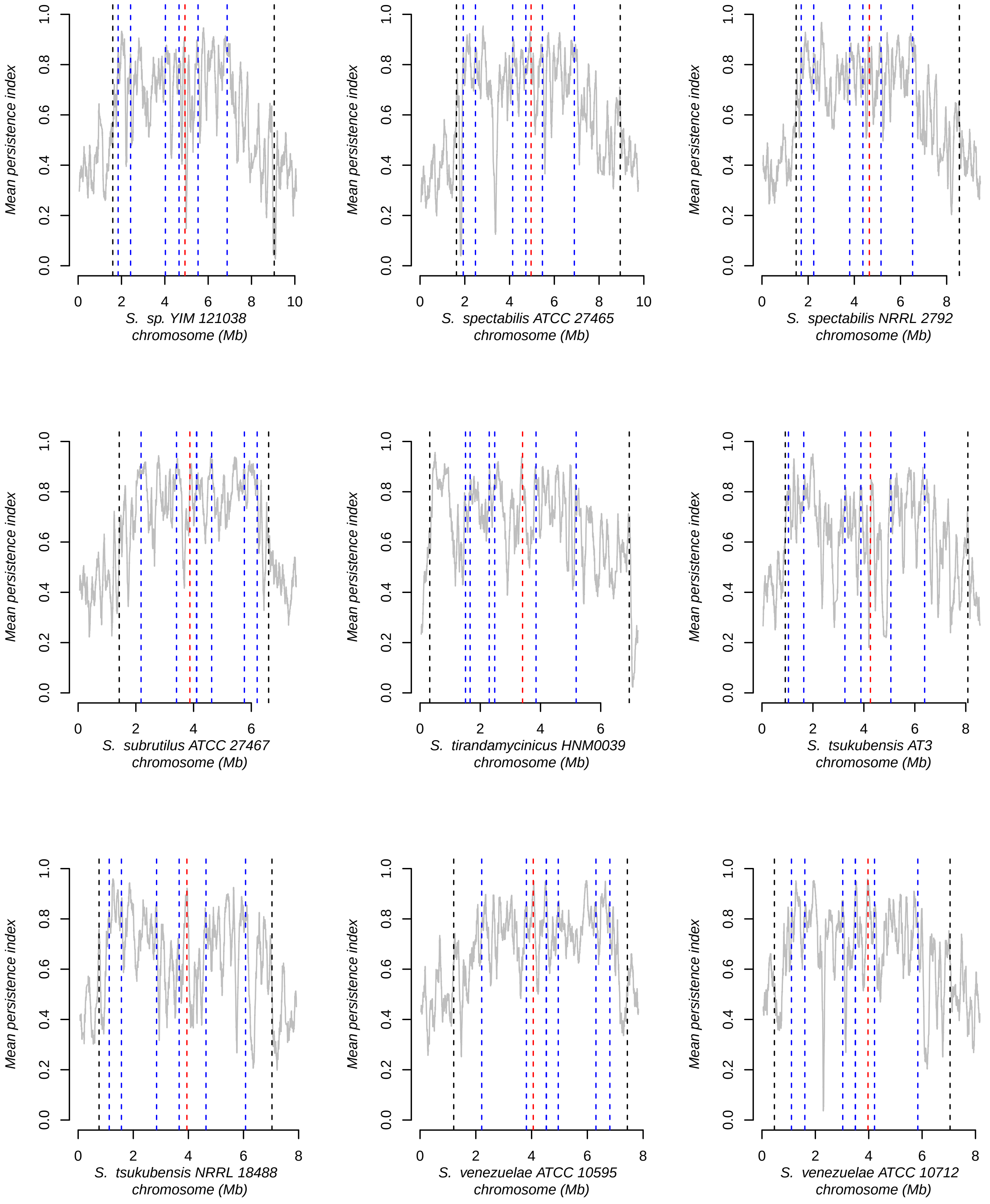

Figure S5 (14/15)

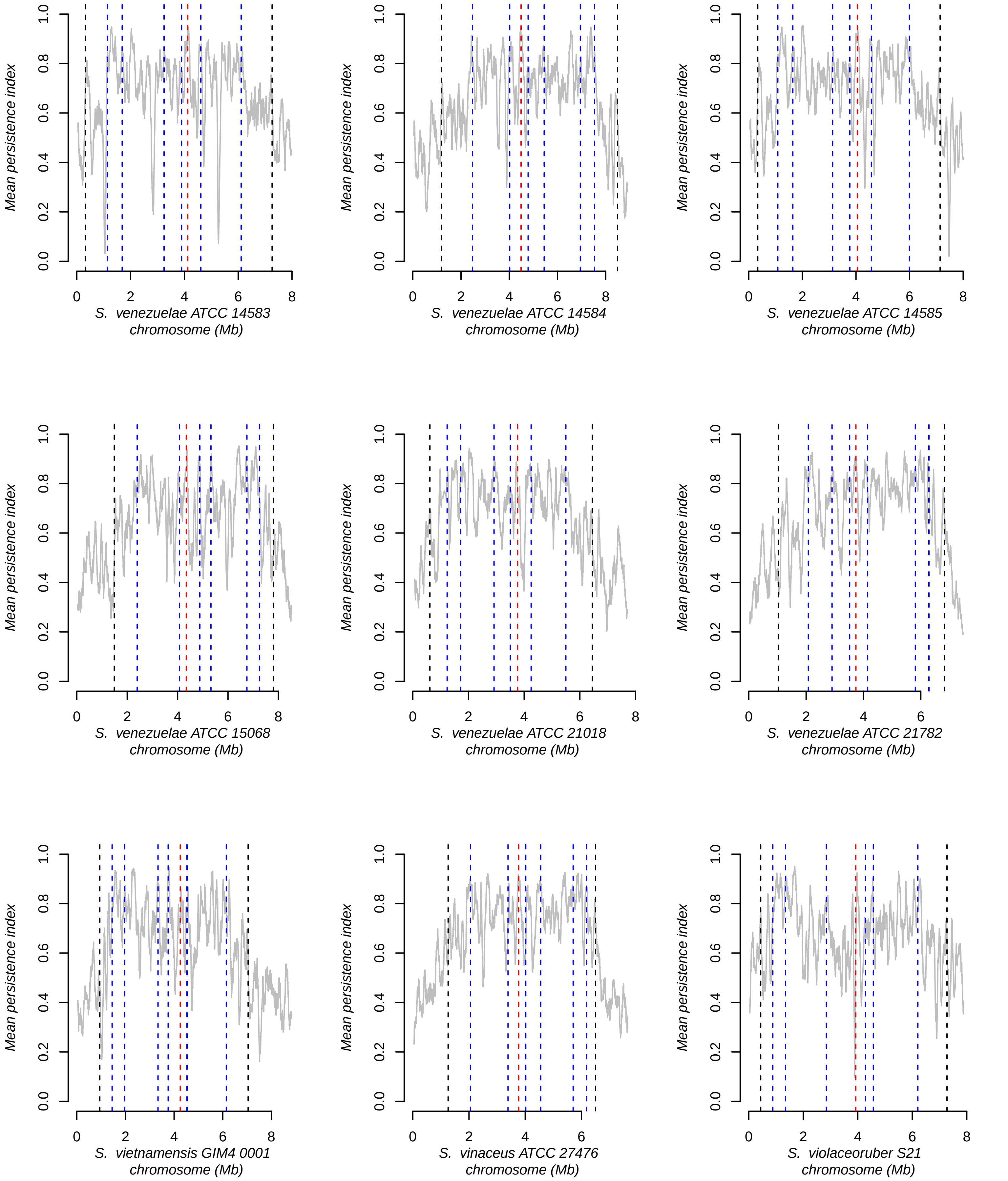

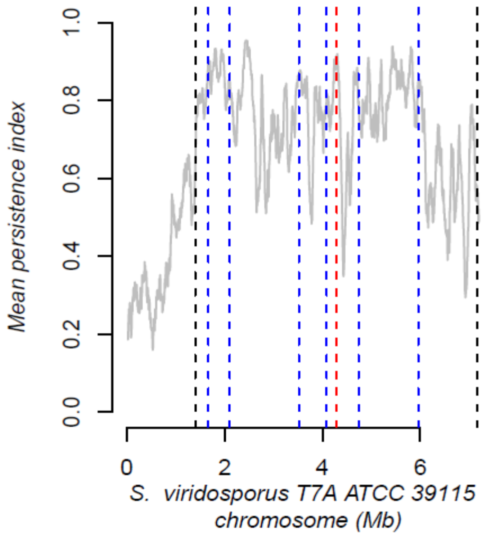

Figure S5: Gene persistence along the chromosomes of 127 *Streptomyces* strains/species

The level of persistence (*y*-axis) along the chromosome (*x*-axis) is represented using a sliding window [81 coding sequences (CDSs), with 1 CDS steps]. The positions of the most external core genes, all *rrn* operons and the origin of replication are indicated by black, blue and red dashed lines, respectively. The sequences released from the databases (**Supplemental Table S1**) were directly analyzed, without changing their orientation. To decide whether a sequence from the database is in the same orientation as the reference consensus used in this study (*Streptomyces viridosporus* T7A ATCC 39115), it is necessary to consider the results of pairwise comparison presented in **Supplemental Figure S2**: when the diagonal starts at the bottom left and ends at the top right, it means that the genome sequence available in the databases is oriented as in the consensus, and that the *rrn* configuration shown below each graph can be directly transposed onto the graph. If not, the sequence is in the opposite direction. In this case, the consensus should be reversed when transposed on the graph (e.g. '*rrn* ABCDEF *dnaA*+' becomes '*rrn* F*E*D*C*B*A* *dnaA*-').
