# Supplementary material for: Ribosomal RNA operons define a central functional compartment in the Streptomyces chromosome
Source: Nucleic Acids Res. 2022 Nov 21;50(20):11654–69. doi: 10.1093/nar/gkac1076 (PMC9723626; doi:10.1093/nar/gkac1076)
Supplement: gkac1076_Supplemental_Files [file gkac1076_supplemental_files.zip › Supplementary_tables.zip!/Supplementary_tables/Supplementary_Table_S7.pdf]

Table S7: Remarkable species regarding core gene order compared to the *Streptomyces* consensus

Species with core genes ordered exactly as in the consensus
Streptomyces viridosporus T7A ATCC 39115<br>*Streptomyces* sp. GGCR 6<br>*Streptomyces* sp. QMT 28<br>*Streptomyces* sp. S10 2016<br>*Streptomyces* sp. S1A1 7
**Species with core genes ordered as in the consensus, except for two genes (local inversion)**
Streptomyces ambofaciens ATCC 23877<br>*Streptomyces ambofaciens* DSM 40697<br>*Streptomyces chartreusis* NRRL 3882<br>*Streptomyces coeruleorubidus* ATCC 13740<br>*Streptomyces ficellus* NRRL 8067<br>*Streptomyces galilaeus* ATCC 14969<br>*Streptomyces pactum* ACT12<br>*Streptomyces parvulus* 2297<br>*Streptomyces* sp. CCM MD2014<br>*Streptomyces* sp. Go 475<br>*Streptomyces* sp. SS52<br>*Streptomyces* sp. SYP A7193
